# Quantitation and Distribution of *Epichloë*-Derived Alkaloids in Perennial Ryegrass Tissues

**DOI:** 10.3390/metabo13020205

**Published:** 2023-01-30

**Authors:** Simone Vassiliadis, Priyanka Reddy, Joanne Hemsworth, German C. Spangenberg, Kathryn M. Guthridge, Simone J. Rochfort

**Affiliations:** 1 Agriculture Victoria, AgriBio Centre for AgriBioscience, Bundoora, VIC 3083, Australia; 2 School of Applied Systems Biology, La Trobe University, Bundoora, VIC 3083, Australia

**Keywords:** mycotoxin, *Lolium perenne*, pasture, endophyte, quantitation, indole diterpene, LC-MS

## Abstract

Perennial ryegrass (*Lolium perenne* L.), an economically important pasture and turf grass, is commonly infected with asexual *Epichloë* species endophytes. Endophytes provide enhanced bioprotection by producing alkaloids, and research often focusses on the negative impact on grazing animals. However, alkaloid distribution throughout the plant and their role in biocontrol of insect pests and diseases are less well understood. Additionally, intermediate compounds have not been investigated for their impacts on animal welfare and biological control in pasture-based scenarios. Here, a single liquid chromatography–mass spectrometry (LC-MS) method was used to measure seven alkaloids in different perennial ryegrass tissues infected with SE or NEA12 endophytes. High alkaloid recoveries and a clear plant matrix effect emphasize the importance of using matrix-matched standards for accurate quantitation. The method is sensitive, detecting alkaloids at low concentrations (nanogram levels), which is important for endophyte strains that produce compounds detrimental to livestock. Concentrations were generally highest in seeds, but distribution differed in the shoots/roots: peramine, terpendole E, terpendole C and lolitrem B were higher in shoots, whilst ergovaline, paxilline and epoxy-janthitrem I were more evenly distributed throughout the two tissues. Knowledge of alkaloid distribution may allow for concentrations to be predicted in roots based on concentrations in the shoots, thereby assisting future determinations of resistance to insects, especially subterranean root-feeding pests.

## 1. Introduction

*Epichloë* fungal endophytes of perennial ryegrass (*Lolium perenne* L.) and tall fescue (*L. arundinaceum*) have been commercially exploited to benefit the pasture and turf industries [1,2,3]. They provide better persistence through improved tolerance to drought and mineral stress, and the production of endophyte-derived alkaloids can confer resistance to phytopathogens, insects and animal herbivores [4,5,6,7,8,9]. The natural association of perennial ryegrass infected with *Epichloë* is commonly studied due to its agricultural importance. Standard toxic endophyte (SE), a strain of *E. festucae* var. *lolli*, produces ergovaline, lolitrem B and peramine. Peramine is known to be active against the insect pasture pest the Argentine stem weevil (*Listronotus bonariensis*), yet its production has no known toxic effects on grazing livestock [10,11,12,13]. Ergovaline and the indole diterpene lolitrem B also provide protection against pasture pests, and these compounds are toxic to grazing livestock. This has led to the search for novel endophyte strains that do not produce lolitrem B, such as NEA2 and AR1, which are also in use commercially. Endophyte strains with novel chemistry include the *Lp*TG-3 strains AR37 and NEA12, which produce epoxy-janthitrems, a group of indole diterpenes with structural similarities to lolitrem B. Tremors and staggers are less severe in livestock feeding on AR37 pastures compared to SE pastures, suggesting that, whilst epoxy-janthitrems may be toxic, they are not as potent as lolitrem B [14,15,16,17]. Epoxy-janthitrem I (11,12-epoxy-janthitrem G) is thought to be the major janthitrem produced by perennial ryegrass endophytes [18], and its production is often linked to the insecticidal activities of pasture pests [19,20]. The importance of epoxy-janthitrems for livestock and insects has been tested; purified epoxy-janthitrems induce tremors in mice, and the weight and feeding of porina larvae (*Wiseana cervinata*) are negatively affected [19]. Although the bioprotective impacts of endophyte-derived alkaloids have been described, there is a knowledge gap regarding key intermediate compounds associated with end-product indole diterpene alkaloids as well as their in planta distribution and potential for bioprotection in roots.

Research suggests that janthitrems share a common biochemical pathway with lolitrem B. Lolitrem B and the precursory intermediate alkaloids paxilline and terpendole C induce tremors in grazing animals; paxilline may also be a precursor to janthitrem production [18,21,22,23]. Non-tremorgenic intermediates are associated with the production of toxic end-product alkaloids. For example, terpendole I is a precursor to lolitrem B in *Epichloë* endophytes [24], whilst paspaline is a key intermediate prior to the splitting of two pathways, one ending with lolitrems and another ending with the production of janthitrems [25]. 

Forage improvement programs have focused on selecting novel endophytes that do not negatively impact livestock; however, it is clear that complex mixtures of the indole diterpene intermediate compounds will still be present [26]. The indole diterpene pathway is therefore of particular interest to the pastoral industry because *Epichloë* strains that do not produce the end products (lolitrem B and epoxy-janthitrem I) can still produce toxic intermediate compounds [27]. It is therefore unwise to assign a single compound to a tremorgenic disease response.

Typically, it is the leaves and pseudostems, approximately 5 cm above the crown, of endophyte-infected perennial ryegrasses that are screened for major compounds, as it is these on which animals graze. Such analysis is essential for determining potential pasture toxicity as well as evaluating novel endophyte strains prior to commercialization. However, the distribution of endophyte-derived alkaloids, both end products and intermediates, throughout the host plant is of equal importance, and this has not yet been investigated. 

Alkaloid concentrations in ryegrass seeds are generally higher than those observed in other tissues, and for this reason seeds are used for extraction and purification purposes [28,29,30]. Alkaloids in the leaves and pseudostems may also protect against insect pests. Common pasture and turf pests in Australia include the African black beetle (*Heteronychus arator*), the Argentine stem weevil and the redheaded pasture cockchafer (*Adoryphorus coulonii*), amongst others [31]. Meanwhile, knowledge regarding alkaloid concentrations in the roots is limited, and such information is required to determine how effective insecticidal alkaloids are for the control of subterranean pests. For example, the pasture root aphid (*Aploneura lentisci*) is an agriculturally important insect pest of perennial ryegrass which infests and feeds on roots and thereby reduces plant growth [32,33]. Pot trial studies have shown that pasture root aphids are negatively impacted when feeding on the roots of AR37- and SE-infected plants [34]. The study suggests that ergovaline and epoxy-janthitrems may be responsible for reduced aphid numbers; however, the exact mechanisms involved in insect resistance in roots are not known, nor are the levels required for bioprotection in roots [34]. Analysis of alkaloids in the roots, as well as the shoots, is therefore highly desirable and would provide valuable information for the future evaluation of *Epichloë*-mediated insect pest protection in pasture and turf.

This study employs a robust liquid chromatography–mass spectrometry (LC-MS) method to quantitate seven agriculturally important alkaloids, including indole diterpene intermediates and end products: peramine, ergovaline, lolitrem B, paxilline, terpendole E, terpendole C and epoxy-janthitrem I in SE- and NEA12-infected seeds, shoots and roots. The study aims are two-fold: to accurately measure and compare the alkaloids in different plant matrices and to determine their distribution throughout the host plant, with a focus on the shoots versus the roots.

## 2. Materials and Methods

### 2.1. Chemicals

The extraction solvent (methanol, >99.9% pure) and the mobile phase solvents (acetonitrile with 0.1% formic acid, >98.5% pure, and water with 0.1% formic acid) were purchased from Fisher Chemical, Fair Lawn, NJ, USA. 

The alkaloid standards used in the study were peramine hemisulfate (Toronto Research Chemicals, North York, ON, Canada), ergotamine D-tartrate (>97% pure), paxilline (>98% pure), terpendole E (>95% pure) and terpendole C (>95% pure) (Sigma-Aldrich, St. Louis, MO, USA) and janthitrem A (>95% pure, BioAustralis, Smithfield, NSW, Australia). Lolitrem B was isolated from perennial ryegrass seeds [30].

### 2.2. Plants and Alkaloid Extractions

Perennial ryegrass seeds (*Lolium perenne* L.) were grown in 10 cm pots filled with potting mix containing 30 L of seed raising mix (Van Schaik’s Biogro, Dandenong South, VIC, Australia) with 0.9 L of fine vermiculite, 0.6 L of fine perlite, 25 g of nutricote, 20 g of water-holding granules and 2.5 g of trace elements. Plants were grown to maturity, split, and repotted in a glasshouse controlled at 25 °C, 60% relative humidity, with natural lighting (approximately 16 h photoperiod). The seeds (cultivar Alto) were supplied by Barenbrug (Christchurch, New Zealand) and hosted either standard toxic endophytes (SE) or NEA12. Seeds were also supplied without endophytes (WE).

Five plants were harvested per cultivar–endophyte association. Plants were removed from their pots and soil was washed from the roots. Sharp scissors were used to separate the shoots (leaves and pseudostems, 5 cm from the base of the plant) from the roots. Tissues were immediately transferred to paper bags and stored at −80 °C until freeze-drying for approximately 48 h (Alpha 1–4 LD plus, Christ, Germany). Seeds (3 g) were not freeze-dried. All tissues were ground at 1500 rpm until a fine powder was formed (Genogrinder 2010, SPEX SamplePrep, Metuchen, NJ, USA), and the replicates were pooled. 

Alkaloids were extracted according to Vassiliadis et al. [35]. In summary, 20 mg (±0.2 mg) of tissue was extracted twice with 1 mL of 80% methanol (methanol and milli-Q water, 80:20, *v*:*v*), followed by mixing (multi tube vortex mixer, MTV1; Ratek, VIC, Australia), sonication (250TD; SoniClean, SA, Australia) and spinning (161,000 g, 21 °C, Eppendorf, 5415D) for 5 mins each. The extract was transferred to a 2 mL HPLC vial.

### 2.3. Calibration and Method Validation

Validation parameters were followed according to international guidelines [36]. The linear range of alkaloids was determined using a mixed stock of seven alkaloid standards, diluted from approximately 1000 ng/mL to 0.5 ng/mL (Table 1). Linear regression models were constructed from the neat standard in 80% methanol:water (*v*:*v*) following eight injections of standards 1–10. The mixed standards at levels four (~10 ng/mL), seven (~100 ng/mL) and ten (~1000 ng/mL) were used to assess alkaloid recoveries (REs) and matrix effects (MEs). These concentrations were employed to represent low, medium and high levels relative to the calibration curve. 

The limit of detection (LOD) and limit of quantitation (LOQ) were determined by measuring the signal-to-noise ratio (S/N), where S/N > 3 and S/N > 10, respectively [36]. The standards at low, medium and high concentrations were used to create calibration curves and measure the accuracy and precision of the method. Peak areas based on the molecular [M+H]^+^ ion were used for all calculations, and the curves were adjusted by excluding high-concentration points to avoid overfitting of the data at very low concentrations, i.e., near the lower LOQ. Accuracy assessed the percent deviation between the measured concentration and the expected (actual) concentration. Precision was determined by measuring the %RSD of the peak response. Selectivity was assessed by visually inspecting the Alto-WE samples spiked with alkaloids and comparing them with the chromatograms of non-spiked samples. The presence of interfering compounds from within the plant matrix was established.

Plant ME and RE was established by spiking the tissues (shoots, roots and seeds) at the three concentration levels (low, medium and high), using the equations described by Vassiliadis et al. [35]. ME was calculated by comparing the peak response of the post-extracted spiked samples (roots, shoots or seeds) with the neat standard at the same concentration. An ME of <100% indicated ion suppression, whilst an ME of >100% indicated ion enhancement. REs of the analytes were established by comparing the peak responses of pre-extracted spiked samples with post-extracted spiked samples. Full recovery was determined at 100%. The presence of endogenous alkaloids in non-spiked samples was subtracted from the equation.

### 2.4. LC-MS Paramaters

LC-MS parameters were followed according to Vassiliadis et al. [35]. Alkaloids were analyzed using a Vanquish ultra-high-performance liquid chromatography (UHPLC) system (Thermo Fischer Scientific, Bremen, Germany) coupled to a Thermo Fisher Q Exactive Plus mass spectrometer (QE MS) (Waltham, MA, USA; Thermo, Bremen, Germany). Separation of the analytes occurred on a 150 mm × 2.1 mm Thermo Hypersil Gold, 1.9 µm HPLC reverse-phase column with a gradient mobile phase of A, 0.1% formic acid in water, and B, 0.1% formic acid in acetonitrile. The starting conditions were 2% B, followed by a linear gradient to 100% B (0–11 min), a hold for 4 mins (11–15 min) and then a return to the initial conditions. The total run time was 20 mins at a flow rate of 0.3 mL/min. The column compartment was set to 30 °C. The sample (3 µL) was injected and analyzed in positive electrospray ionization mode using full scan with a range of 80–2000 *m*/*z* and with the resolution set at 35,000. The MS source heater temperature was set to 310 °C; the heated capillary was 320 °C. Nitrogen was used as the sheath, auxiliary and sweep gases, which were set to 28, 15 and 4 L/min, respectively, and the spray voltage was 3.6 kV. Identification of compounds was performed via MS1 (Supplementary Figure S1), whilst ergovaline and epoxy-janthitrem I were established using a selection of Alto-SE and Alto-NEA12 samples and identified based on their MS/MS (MS2) product ion patterns (Supplementary Figure S2) and comparisons with the available literature [18,37]. The collision energy was 35 V, and the maximum ion time was 200 milliseconds. Calibration of the instrument occurred prior to analysis using Pierce LTQ Velos ESI Positive Ion Calibration Solution (Thermo Scientific, product no. 88323). 

The data were acquired using the Thermo Xcalibur Qual Browser v.3.0.63 (Thermo Scientific, Waltham, MA, USA). The targeted ions were extracted from the full-scan chromatogram, integrated, then quantitated using LCquan v.2.7.0.21 (Thermo Scientific, Waltham, MA, USA). Mass accuracies were checked in neat standards and in the different plant matrices spiked with high or low concentrations, using a selection of Alto-WE samples. These were determined at 3 ppm (parts per million) or better compared to the theoretical mass. 

### 2.5. Alkaloid Quantitation

Alkaloid concentrations were measured in 20 mg samples of roots, shoots and seeds of pooled ryegrass tissues (n = 5), extracted as above. Internal standards were prepared using the low, medium and high concentration levels in post-spiked matrices using Alto-WE root, shoot and seed tissues. Quantitation of alkaloids were compared using either the same or different in-matrix standards. Ergotamine, an ergopeptine that is not naturally produced by *Epichloë* endophytes [38], was used to quantitate ergovaline. Meanwhile, janthitrem A, formally termed 11, 12-epoxyjanthitrem B, was used to quantify epoxy-janthitrem I based on structural similarity (sharing an epoxide group analogous to the epoxy-janthitrems) [19]. Values for ergovaline and epoxy-janthitrem I were recovery-corrected using post-spiked ryegrass samples, i.e., the average quantitative values for ryegrass shoots, roots and seeds spiked with approximately 100 ng of ergotamine or janthitrem A. The differences in molecular weight were adjusted by multiplying the quantitation values by the ratios of the target compounds and the proxies. Alkaloid means, SDs, %RSDs and single-factor ANOVAs were performed using the Real Statistics Resource Pack software (release 7.6) [39]. Alkaloid distribution was calculated as the difference in concentration (%) in the roots versus the shoots.

## 3. Results and Discussion

**Figure 1:**
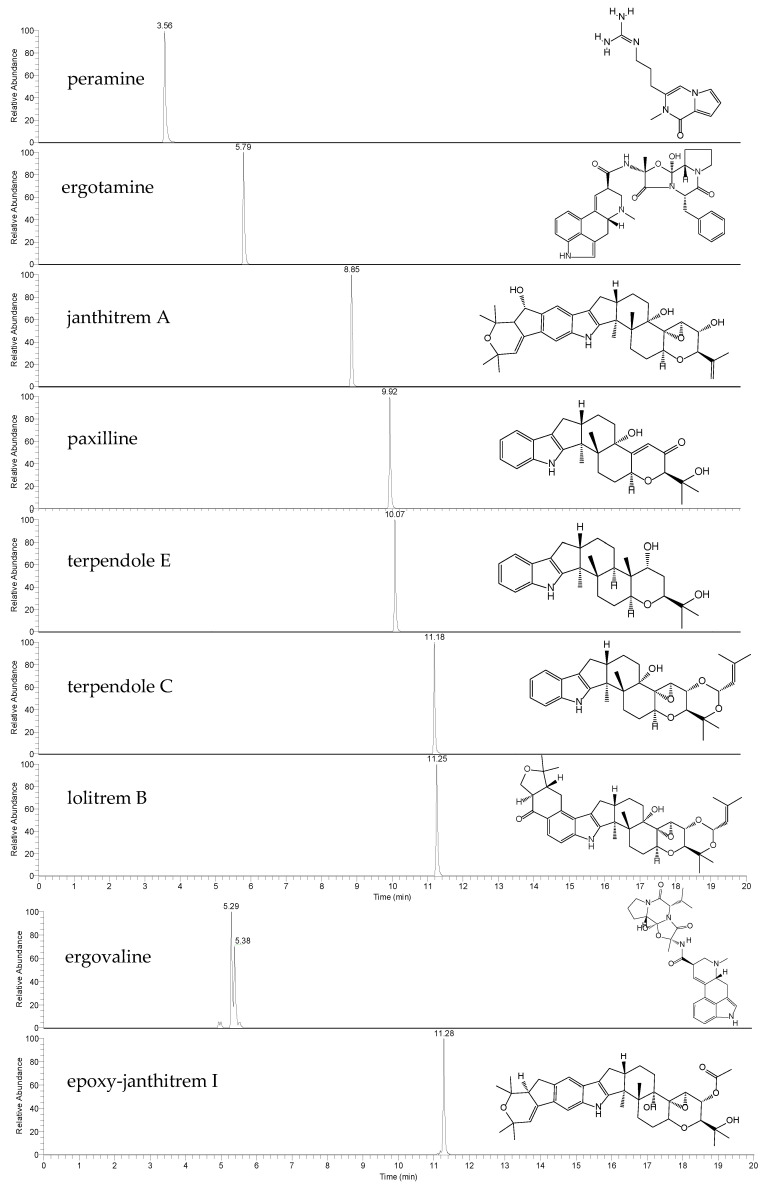
Extracted ion chromatograms (EICs) and chemical structures of the alkaloid standards peramine, ergotamine, janthitrem A, paxilline, terpendole E, terpendole C, lolitrem B, ergovaline and epoxy-janthitrem I. Endogenous ergovaline was extracted from perennial ryegrass Alto-SE seeds, whilst endogenous epoxy-janthitrem I was extracted from Alto-NEA12 seeds.

This work first describes method-validation parameters for the accurate quantitation of seven endophyte-derived alkaloids. The high-resolution LC-MS method provides well-resolved chromatographic peaks (Figure 1). Two co-eluting peaks were observed in the extracted ion chromatogram (EIC) of ergovaline, likely due to isomerization. It is not uncommon for ergot alkaloids to undergo epimerization, a phenomenon which may be influenced by light, heat and stability in solution [40,41,42]. Due to this, the peak areas of both ergovaline and its isomer (likely ergovalinine) were used for quantitation [38]. 

Molecular mass [M+H]^+^, retention time (RT), LOD, LOQ, linearity, accuracy (bias) and precision are reported for each of the alkaloids in Table 2. Detected masses were within 3 ppm of the theoretical mass. The LOD for all alkaloid reference standards was 0.2 ng/mL, whilst the LOQ was 0.5 ng/mL for peramine, ergotamine, terpendole E and lolitrem B and 0.6 ng/mL for janthitrem A, paxilline and terpendole C. All calibration curves were linear and returned *R*^2^ values above 0.99.

Accuracy of quantitation for all compounds was high, between 90–100%, across all concentration levels, except for terpendole C, for which accuracy was 20.2% at 2 ng/mL. Precision was confirmed for all alkaloids, as indicated by relative standard deviation (RSD) values of less than 7.3%. Again, terpendole C was an exception at 66.8% RSD at the lowest measured concentration (2 ng/mL) compared to the actual concentration (10 ng/mL) (Table 2). 

**Figure 2:**
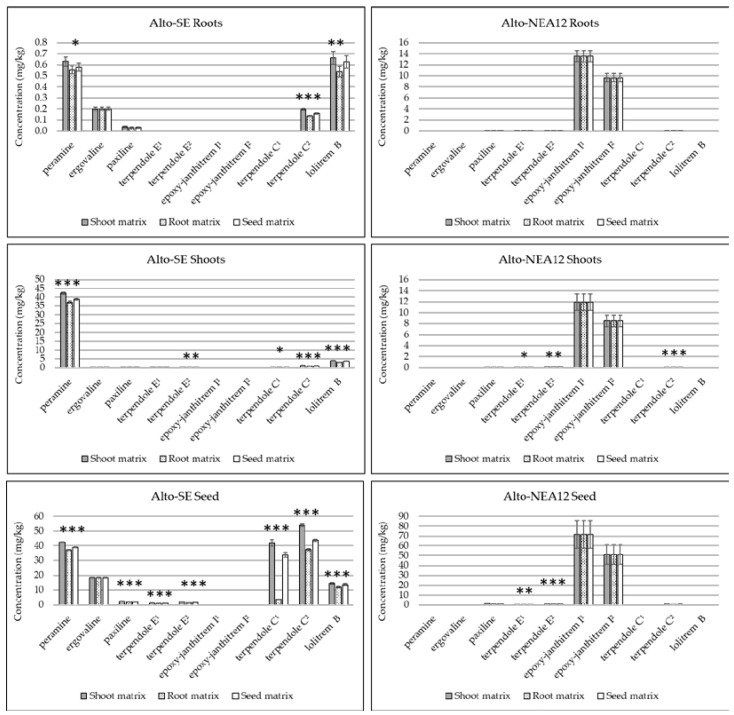
Comparison of alkaloid concentrations (mg/kg) in different perennial ryegrass tissues (pooled, n = 5) quantitated using either the same or different in-matrix standards (root, shoots or seeds). Error bars represent standard errors of the means (SEMs). Asterisks denote significance for each alkaloid according to single-factor ANOVA (* *p* <0.05; ** *p* <0.01; *** *p* <0.001). Denotation of alkaloids as ‘^1^’ indicates molecular ion in the MS1 spectrum; denotation of alkaloids as ‘^2^’ indicates fragment ion in the MS1 spectrum.

Peters et al. suggested that the acceptable criteria for compound accuracy and precision should be within ±15% of the nominal value or ±20% at the lower limit of quantitation (LLOQ) [36]. Closer inspection of the mass spectrum (MS1) data revealed that the molecular ions of terpendole C (*m*/*z* 520.3053), janthitrem A (*m*/*z* 602.3461) and terpendole E (*m*/*z* 438.2995) had lower abundances compared to fragment ions at *m*/*z* 436.2481, 526.2939 and 420.2894, respectively (Supplementary Figure S1). Further investigation of the suitability of the method for these alkaloids were therefore required, as the discrepancy was assumed to be due to in-source fragmentation. As accuracy and precision data for all other alkaloid standards were well within the acceptable limits, the masses of both molecular ions (denoted as janthitrem A^1^, terpendole E^1^ and terpendole C^1^) and fragment ions (denoted as janthitrem A^2^, terpendole E^2^ and terpendole C^2^) for these three standards were assessed for all further validation work (Table 2 and Table 3, Figure 2). Consequently, an accuracy of 96.7% for terpendole C^2^ (the fragment ion) at the low concentration resulted, well within the limit suggested by Peters et al. [36].

No evidence of co-elution of endophyte-derived compounds was observed in spiked and non-spiked perennial ryegrass samples, establishing the selectivity of the method. However, the mass spectral data were very complex, and interference due to other ions was inevitable [35]. Ion interference, a well-known phenomenon, is mainly dependent on the sample matrix [36] and can impact the accurate quantitation of target compounds. This is especially important when measuring agriculturally important toxins in different plant tissues.

### 3.1. Alkaloid Recovery and the Impact of Plant Matrix

Accurate quantitation relies on the analysis of compound REs and potential MEs, particularly when comparing concentrations in different plant tissues. This is especially important for low-abundance toxic alkaloids and their precursors. Here, the REs and potential MEs in the roots, shoots and seeds of perennial ryegrass plants without endophytes (Alto-WE) and with endophytes (Alto-SE and Alto-NEA12) were established (Table 3, Supplementary Table S1). These two strains were selected to represent the suite of known key alkaloids produced by perennial ryegrass–endophyte symbiotes, peramine, ergovaline, lolitrem B (produced by SE) and epoxy-janthitrem I (produced by NEA12).

In general, an ME was established mostly in the form of ion suppression (ME values <100%) (Table 3). This has been previously documented for peramine (67–81%), lolitrem B (67–88%) and ergotamine (77–88%) in perennial ryegrass leaves, matched with high RE rates (77–90%) [35]. The MEs for terpendole C^1^ were variable, and greater consistency was established for those of terpendole C^2^. High MEs (>100%) indicate ion enhancement. These were noted in samples spiked at low concentrations and may have been due to how well the compounds ionized. Quantitatively, these samples may also have been close to the LLOQ, and investigation of the acceptance of their reproducibility, by examination of the %RSD values, is discussed below. 

The REs of all spiked compounds were moderate to high, and results were generally consistent across the different tissues, irrespective of endophyte association (Table 3). In previous studies, RE results were determined for ergotamine from tall fescue grass tillers (77–86%) [43,44], whilst the REs of lolitrem B were much lower in perennial ryegrass seeds (15–34%) [28,29,30]. The REs of the alkaloids with larger fragment ions in the mass spectra, as opposed to the molecular ions, were generally consistent, except for terpendole C^1^, and better RE results were established when quantitation was performed using terpendole C^2^. These results were within the acceptance criteria, between 15–20% RSD, established by Peters et al. [43]. The %RSDs of peak area responses, used to calculate the REs, were low (1–15% RSD), indicating good reproducibility (Supplementary Table S1). Values greater than 15% were typically seen either in perennial ryegrass with low levels of endogenous compounds or samples with low pre- or post-spikes. Furthermore, analysis of the molecular and fragment ions of epoxy-janthitrem I, terpendole E and terpendole C were useful in determining the MEs and REs of these alkaloids. The MEs, REs and %RSDs of epoxy-janthitrem I and terpendole E were similar, so the molecular ion may be used for all further quantitation work. Conversely, the ME and RE results differed for terpendole C. Here, the fragment ion produced the most consistent results, indicating that the fragment ion should be used for future quantitation work using the method described in this study.

### 3.2. Quantitation and Distribution of Alkaloids

Alkaloid concentrations were compared in the roots, shoots and seeds of perennial ryegrass without endophytes (Alto-WE) and with two genetically distinct endophyte strains (Alto-SE and Alto-NEA12) using either the same or different matrix-matched standards (Figure 2, Supplementary Table S2). Comparison of low and high spikes resulted in mass accuracies which were generally below 1 ppm, irrespective of tissue type (Supplementary Table S3). Targeted alkaloids were not observed in Alto-WE plants, whilst endogenous compounds were detected in Alto-SE and Alto-NEA12 tissues, as indicated in the EIC of a representative seed sample (Supplementary Figure S3). 

Quantitation using both the molecular and fragment ions were established for terpendole E and epoxy-janthitrem I, whilst terpendole C was measured in all tissues using the fragment ion only (Figure 2). This confirmed that the fragment ion (terpendole C^2^) is required for quantitation purposes, whilst the molecular ions should be used to quantitate terpendole E and epoxy-janthitrem I. Clearly, the utilization of different matrices impacted the quantitative results, and alkaloid response was greater when measured using analytes spiked in shoots, followed by those spiked in seeds, then those spiked in roots. Differences are likely due to ion enhancement and/or suppression, as mentioned previously. The same quantitative values for ergovaline and epoxy-janthitrem I were established across the different tissues, as both compounds were recovery-corrected using internal post-spiked samples. This is appropriate when chemical standards are not available and the compound is not endogenous in the sample matrix.

Seeds contained the greatest concentrations, followed generally by the shoots and then the roots. Earlier work established 75% of total plant peramine levels and greater concentrations of lolitrem B in the seeds of perennial ryegrass compared to other tissues [28,29,45]. Even in a germinated seedling, the seed mass retains much of the alkaloid content [46]. Such high concentrations allow for the extraction and purification of targeted alkaloids [28,29,30] and may improve seed quality and seedling establishment by protecting against insect herbivory [47]. 

In mature plants, endophyte mycelia (the living fungal tissue) are concentrated in the pseudostems, so alkaloids must be transported from the pseudostems to the rest of the plant. Consequently, concentrations of endophyte-derived alkaloids are highest in pseudostems, and abundance in roots is generally expected to be lower than in the leaf blades [34,35,36,37,38,39,40,41,42,43,44,45,46,47,48]. Due to their role in protecting pasture and turf from insect pests and the implications for the welfare of grazing livestock, the presence and abundance of alkaloids in the shoots has been investigated in depth. In contrast, knowledge is limited regarding the root tissues, for which endophyte-derived alkaloid bioprotection from subterranean pests is critically important for pasture persistence. 

**Figure 3:**
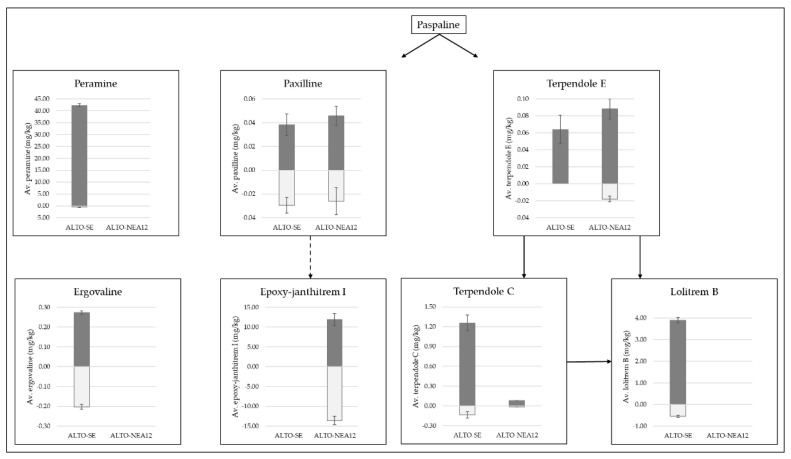
Distributions of peramine and ergovaline alkaloids as well as intermediates and end products of the indole diterpene pathway (paxilline, epoxy-janthitrem I, terpendole E, terpendole C and lolitrem B), as measured in Alto-SE- and Alto-NEA12-infected perennial ryegrass shoots (dark-grey bars) and roots (light-grey bars). Concentrations were measured using the same matrix-matched standards. Solid arrows represent clear pathways according to KEGG metabolic pathway analysis [51,52,53], whilst the dashed arrow represents the tentative pathway to epoxy-janthitrem I synthesis according to Ludlow [18].

Peramine was almost exclusively present in the shoots (99% difference compared to the roots) of Alto-SE (Figure 3), and similar findings have been reported previously [45,48,49]. Peramine is not toxic to grazing livestock, although it is active against some insect pests, with levels greater than 15 ppm reported to protect pastures from the Argentine stem weevil [10,11,12,13,50]. Since levels appear to be low in the roots, peramine is unlikely to contribute to protection from root-feeding insects. 

Ergovaline was more evenly distributed throughout the tissues (26% reduction in roots). While ergovaline is toxic to grazing livestock, the concentrations of ergovaline required to enhance bioprotection in pasture and turf have not been described. However, it does appear that low, sub-clinical levels of ergovaline provide sufficient bioprotection against both above- and belowground pests. It will be important to clearly determine whether the approximate 3:1 shoot:root distribution of ergovaline observed in this study using SE is also the case for other ergovaline-producing strains utilized in pastures, such as NEA2.

Figure 3 also highlights the distribution of intermediate alkaloids associated with the indole diterpene pathway, leading to the end products lolitrem B and epoxy-janthitrem I [18,51,52,53]. The pathway begins with paspaline, but it was not directly measured due to the lack of an available chemical standard. Although it is not toxic or tremorgenic to mammals [54], paspaline is an important intermediate in the biosynthesis of indole diterpenes, as endophyte strains that do not synthesize paspaline fail to produce any tremorgenic indole diterpenoids [55].

The first branch of the pathway leads to the biosynthesis of lolitrem B via the intermediate compounds terpendole E and terpendole C [52,53]. Terpendole E was present in low abundances in the shoots of Alto-SE and was not detected in the roots. Terpendole E was detected in very low abundances in both tissue types from Alto-NEA12 symbiotes; however, levels were reduced by 80% in the roots. Overall concentrations of terpendole C were much lower in Alto-NEA12-infected plants compared to Alto-SE-infected plants, yet similar reductions in the root tissues were determined (86 and 89%, respectively). Likewise, levels of lolitrem B were reduced by 86% in the roots of Alto-SE compared to the shoots. 

Although terpendole E has been identified as non-tremorgenic [55], its role in synthesizing tremorgenic terpendole C and lolitrem B is of interest. In a review article, Eady et al. noted that a non-lolitrem-producing endophyte strain (NEA3) caused ryegrass staggers in livestock [56]. The perennial ryegrass-NEA3 symbiotes contained higher levels of paxilline and terpendole C compared to perennial ryegrass-SE in the same study, indicating that these compounds may have been responsible. Both paxilline and terpendole C are known to causes tremors [23]. Levels as low as 4–8 mg/kg paxilline and 4 mg/kg terpendole C have been shown to induce tremors in mice [26,57]. The roles of these compounds in protection against insect pests are therefore of interest.

Higher concentrations of lolitrem B observed in shoots compared to roots were consistent with previous findings [48]. Pot trial studies revealed that lolitrem B-producing endophytes had no impact on root aphid populations [34]; however, further investigation of the concentrations of these alkaloids in the roots of other symbiotes is required to test this hypothesis. It will be of interest to determine whether the presence of terpendole E, terpendole C and lolitrem B at concentrations approximately one-fifth of those found in aboveground tissues is sufficient to impact subterranean insect pests. 

The second branch of the indole diterpene pathway leads to the biosynthesis of epoxy-janthitrem I via paxilline, and key findings determined that both alkaloids measured were more evenly distributed throughout the plant tissues compared to components of the lolitrem B pathway (Figure 3). Compared to the shoots, paxilline was reduced by 23% in the roots of Alto-SE and by 43% in the roots of Alto-NEA12. In contrast, epoxy-janthitrem I was increased by 14% in the roots of Alto-NEA12 compared to the shoots. Paxilline is a proposed precursor to janthitrem production [18]. In a recent study on the genetics of epoxy-janthitrem biosynthesis, paxilline and epoxy-janthitrems I-IV were measured in planta following knockdown of the *jtmD* gene. Paxilline levels were significantly reduced by 40–55% when moderate reductions in epoxy-janthitrems I-IV were observed, while 82% reductions in epoxy-janthitrems I-IV did not impact paxilline levels, leading the authors to suggest an overaccumulation of the precursor compound [18]. In the current study, low levels of paxilline may be due to the utilization of the compound for the synthesis of epoxy-janthitrem I. 

Such information is crucial for understanding the role of alkaloids in the biocontrol of pasture pests. For example, the AR37 endophyte strain confers resistance to a wide range of insect pests, including root aphids and porina (*Wiseana* spp.) [32,33,58,59], and, whilst the mechanisms of resistance are unknown, the production of epoxy-janthitrems are thought to be responsible [20,34]. It will be of interest to determine whether the presence of paxilline also provides some protection against pests. 

This study established that paxilline and epoxy-janthitrem I are more evenly distributed between the shoots and roots compared to lolitrem B, terpendole E and terpendole C. The epoxy-janthitrems are lipophilic compounds that are not easily translocated around different plant parts. Therefore, the way in which epoxy-janthitrem I accumulates in the roots has yet to be determined. Nonetheless, the distributions of epoxy-janthitrem I and paxilline throughout the plant are of interest, as high levels in shoots reflect similar levels in the roots.

## 4. Conclusions

The accurate quantitation- and distribution- of *Epichloë*-derived alkaloids in different plant tissues are crucial for understanding the key role alkaloids play in bioprotection against insect pests as well as their impacts on the welfare of grazing mammals. The LC-MS method described here is sensitive enough to measure alkaloids even when they are present at very low concentrations. This becomes important when screening endophytes for their quantitative alkaloid profiles, especially when some compounds, such as paxilline and terpendole C, are highly tremorgenic and have thus far been ignored in the literature as agents of ryegrass staggers. Key findings revealed that ergovaline, paxilline and epoxy-janthitrem I are more evenly distributed in the shoots and roots compared to peramine, terpendole E, terpendole C and lolitrem B, which are more abundant in the shoots. The ability to accurately measure alkaloid presence in roots, or indeed predict abundance based on expected ratios, enables knowledge-based development of perennial ryegrass–endophyte associations with root insect pest resistance, thereby offering enhanced persistence and sustainable pastures. It would be advantageous to investigate this theory in future studies using an array of different perennial ryegrass–endophyte symbiotes.

## Figures and Tables

**Table 1:** The linear range of alkaloid standards (ng/mL).

Standard	1	2	3	4 *	5	6	7*	8	9	10 *
Peramine	0.46	0.91	4.55	9.11	22.77	45.54	91.07	227.68	455.35	910.71
Ergotamine	0.54	1.08	5.41	10.83	27.06	54.13	108.25	270.64	541.27	1082.54
Janthitrem A	0.60	1.20	5.99	11.97	29.93	59.85	119.70	299.25	598.50	1197.00
Paxilline	0.55	1.10	5.50	11.00	27.50	55.00	110.00	275.00	550.00	1100.00
Terpendole E	0.48	0.95	4.75	9.50	23.75	47.50	95.00	237.50	475.00	950.00
Lolitrem B	0.60	1.20	6.00	12.00	30.00	60.00	120.00	300.00	600.00	1200.00
Terpendole C	0.50	1.00	5.00	10.00	25.00	50.00	100.00	250.00	500.00	1000.00

* Standards constitute the low (4), medium (7) and high (10) levels used for matrix spikes.

**Table 2:** LOD, LOQ, linearity, accuracy (bias) and precision for each of the alkaloid standards at low, medium and high concentrations.

Alkaloid	Ion	RT	Formula	Δ ppm	Resolution	LOD/LOQ	Equation	*R* ^2^	Level	Actual Concentration	Measured Concentration	Accuracy	Precision
	*(m*/*z*)	(min)	[M+H]^+^			(ng/mL)				(ng/mL)	(ng/mL)	(% Bias)	(% RSD)
									Low	9.1	10.1	110.9	4.4
Peramine	248.1504	3.56	C_12_H_18_N_5_O	−0.79	34,407	0.2/0.5	y = 571,742x	0.9928	Med	91.1	91.0	99.9	2.4
									High	910.7	910.5	100.0	6.8
									Low	10.8	9.8	90.4	3.5
Ergotamine	582.2708	5.79	C_33_H_36_N_5_O_5_	−0.70	23,707	0.2/0.5	y = 320,514x	0.9983	Med	108.3	108.4	100.1	5.0
									High	1082.5	1085.5	100.3	1.8
									Low	12.0	10.8	90.5	4.7
Janthitrem A ^1^	602.3461	8.85	C_37_H_48_NO_6_	−2.56	23,307	0.2/0.6	y = 90,835x	0.9970	Med	119.7	119.8	100.1	2.4
									High	1197.0	1200.0	100.2	3.6
									Low	12.0	13.4	112.2	5.6
Janthitrem A ^2^	526.2939	8.85	C_34_H_40_NO_4_	−2.35	24,707	0.2/0.6	y = 99,906x	0.9989	Med	119.7	119.6	99.9	3.0
									High	1197.0	1197.8	100.1	2.5
									Low	11.0	10.4	94.5	3.4
Paxilline	436.2478	9.92	C_27_H_34_NO_4_	−1.00	27,407	0.2/0.6	y = 213,433x	0.9975	Med	110.0	110.1	100.1	2.8
									High	1100.0	1101.3	100.1	3.8
									Low	9.5	8.9	93.9	4.3
Terpendole E ^1^	438.2995	10.07	C_28_H_40_NO_3_	−1.76	27,207	0.2/0.5	y = 128,737x	0.9921	Med	95.0	95.1	100.1	3.1
									High	950.0	952.7	100.3	6.6
									Low	9.5	9.4	99.4	3.5
Terpendole E ^2^	420.2894	10.07	C_28_H_38_NO_2_	−0.82	28,007	0.2/0.5	y = 362,759x	0.9980	Med	95.0	95.0	100.0	3.7
									High	950.0	950.2	100.0	3.5
									Low	10.0	2.0	20.2	66.8
Terpendole C ^1^	520.3053	11.18	C_32_H_42_NO_5_	−0.87	24,807	0.2/0.6	y = 362,759	0.9980	Med	100.0	100.8	100.8	4.7
									High	1000.0	1003.2	100.3	6.6
									Low	10.0	9.7	96.7	3.8
Terpendole C ^2^	436.2481	11.18	C_27_H_34_NO_4_	−0.29	27,407	0.2/0.6	y = 44,335x	0.9919	Med	100.0	100.0	100.0	3.7
									High	1000.0	1000.5	100.1	3.9
									Low	12.0	11.5	96.2	7.3
Lolitrem B	686.4049	11.25	C_42_H_56_NO_7_	−0.32	21,607	0.2/0.5	y = 119,033x	0.9971	Med	120.0	120.0	100.0	4.3
									High	1200.0	1200.9	100.1	4.2

Data were collected by duplicate analysis over eight injections. Denotation of alkaloids as ‘^1^’ indicates molecular ion in the MS1 spectrum; denotation of alkaloids as ‘^2^’ indicates fragment ion in the MS1 spectrum.

**Table 3:** The recovery rates (REs, %) and matrix effects (MEs, %) of alkaloid standards spiked at three levels in ground perennial ryegrass shoots, roots and seeds.

			Peramine		Ergotamine		Janthitrem A ^1^		Janthitrem A ^2^		Paxilline		Terpendole E ^1^		Terpendole E ^2^		Terpendole C ^1^		Terpendole C ^2^		Lolitrem B	
Tissue	Plant	Level	RE	ME	RE	ME	RE	ME	RE	ME	RE	ME	RE	ME	RE	ME	RE	ME	RE	ME	RE	ME
	Alto-WE	Low	51	117	81	94	85	104	85	107	90	100	91	103	89	98	94	215	91	95	91	91
		Med	58	113	88	93	92	101	93	103	95	98	97	101	95	98	100	103	95	95	93	97
		High	62	96	88	94	92	93	93	96	94	93	92	94	95	93	95	96	98	93	99	87
Roots	Alto-SE	Low	51	96	81	100	89	102	93	101	90	106	92	102	91	99	110	175	88	101	86	100
		Med	55	98	82	95	91	95	91	100	91	98	92	100	92	96	89	108	93	95	93	98
		High	62	90	84	95	91	89	92	94	92	95	89	96	92	93	92	95	92	92	93	92
	Alto-NEA12	Low	52	101	76	100	90	104	90	109	91	101	88	104	90	100	179	140	88	102	88	98
		Med	59	99	81	96	91	102	91	107	90	101	92	102	89	99	102	103	89	96	91	97
		High	63	91	85	92	93	89	93	97	92	94	93	93	92	93	99	90	95	90	95	91
	Alto-WE	Low	99	88	93	93	96	66	88	77	96	61	96	69	96	66	120	120	96	59	123	61
		Med	96	89	90	88	87	66	85	72	88	62	90	69	91	65	88	61	92	59	94	72
		High	88	85	81	87	64	74	65	80	60	74	64	74	70	74	62	71	63	69	81	81
Shoots	Alto-SE	Low	188	85	87	84	82	72	85	74	95	60	87	67	90	62	139	127	82	71	30	31
		Med	84	74	89	79	77	68	78	72	75	63	77	69	81	66	74	64	76	57	90	73
		High	82	84	77	86	52	75	54	80	50	73	53	73	58	78	46	72	49	71	75	85
	Alto-NEA12	Low	92	97	83	96	78	73	82	79	55	69	75	73	80	68	89	126	83	63	64	31
		Med	94	98	89	87	82	66	81	71	81	62	83	69	84	66	85	61	80	60	92	70
		High	86	91	83	85	59	73	61	78	56	72	59	72	64	76	54	68	57	69	83	78
	Alto-WE	Low	87	104	84	94	87	98	91	97	93	99	92	100	92	92	92	367	91	92	93	87
		Med	94	98	88	88	97	88	95	93	96	94	97	97	101	85	92	99	93	87	94	90
		High	95	92	95	92	90	90	88	94	94	91	97	89	95	90	90	88	93	86	95	86
Seeds	Alto-SE	Low	50	284	87	88	91	105	92	93	78	98	74	112	75	90	37	190	61	71	18	166
		Med	79	102	90	85	93	98	93	93	93	95	92	102	92	87	55	124	43	79	80	86
		High	91	87	90	91	89	90	89	93	89	92	91	92	91	89	84	97	85	83	90	85
	Alto-NEA12	Low	85	100	80	96	85	100	86	97	83	98	79	111	86	89	85	470	80	74	93	85
		Med	87	101	85	86	88	91	87	93	87	95	89	95	88	90	88	97	87	87	91	85
		High	90	92	90	90	86	91	86	93	90	90	89	89	90	89	88	88	90	85	94	83

The concentration levels used for low, medium and high spikes are indicated in Table 1. Denotation of alkaloids as ‘^1^’ indicates molecular ion in the MS1 spectrum; denotation of alkaloids as ‘^2^’ indicates fragment ion in the MS1 spectrum.

## Data Availability

The data presented in this study are contained in the article.

## Supplementary Materials

The following supporting information can be downloaded at: https://www.mdpi.com/article/10.3390/metabo13020205/s1, Figure S1: Positive MS1 spectra for the alkaloids. The spectra show that the fragment ions of *m*/*z* 526 (janthitrem A), *m*/*z* 420 (terpendole E) and *m*/*z* 436 (terpendole C) are of greater abundance compared to the parent ions (janthitrem A, *m*/*z* 602; terpendole E, *m*/*z* 438; and terpendole C, *m*/*z* 520, respectively). Both the parent and fragment ions were used for quantitative studies for comparative purposes.; Figure S2: MS^2^ fragmentation pattern of a) ergovaline and b) epoxy-janthitrem I; Table S1: Recovery and matrix effect data for alkaloids pre- or post-spiked at a low, medium or high concentration in ryegrass shoots without endophyte (Alto-WE) or with endophyte (Alto-SE and Alto-NEA12). The alkaloids are peramine (per), ergovaline (ev), ergotamine (et), janthitrem A (janth A), paxilline (pax), terpendole E (terp E), epoxy-janthitrem I (janth I), terpendole C (terp C) and lolitrem B (lol B). The Mean values (peak area) and standard deviations (SD.) are in arbitrary units, x10^4^. %RSD, percent relative standard deviation.; Table S2: Comparison of the mean alkaloid concentrations in different ryegrass-endophyte associations (Alto-SE, Alto-NEA12) and quantitated using the calibration curves constructed by in-standard matrixes (shoots, roots or seeds).; Table S3: The effect of alkaloid concentration (low or high spikes) and plant matrix (shoot, root or seed) on mass accuracy.; Figure S3: Extracted ion chromatograms (EIC) of alkaloids in neat solution and in Alto-WE, Alto-SE and Alto-NEA12 of a representative seed sample. Arrows show the peak at a given retention time.

## References

[1] Scott B. (2001). *Epichloë* endophytes: Fungal symbionts of grasses. Curr. Opin. Microbiol..

[2] Schardl C.L. (2001). *Epichloë festucae* and related mutualistic symbionts of grasses. Fungal Genet. Biol..

[3] Schardl C.L., Young C.A., Hesse U., Amyotte S.G., Andreeva K., Calie P.J., Fleetwood D.J., Haws D.C., Moore N., Oeser B. (2013). Plant-symbiotic fungi as chemical engineers: Multi-genome analysis of the clavicipitaceae reveals dynamics of alkaloid Loci. PLoS Genet..

[4] Malinowski D., Belesky D. (2019). *Epichloë* (formerly *Neotyphodium*) fungal endophytes increase adaptation of cool-season perennial grasses to environmental stresses. Acta Agrobot..

[5] Saikkonen K., Wäli P., Helander M., Faeth S.H. (2004). Evolution of endophyte–plant symbioses. Trends Plant Sci..

[6] Malinowski D.P., Belesky D.P. (2000). Adaptations of endophyte-infected cool-season grasses to environmental stresses: Mechanisms of drought and mineral stress tolerance. Crop Sci..

[7] Clay K., Schardl C. (2002). Evolutionary origins and ecological consequences of endophyte symbiosis with grasses. Am. Nat..

[8] Zain M.E. (2011). Impact of mycotoxins on humans and animals. J. Saudi Chem. Soc..

[9] Wilson D. (1993). Fungal endophytes: Out of sight but should not be out of mind. Oikos.

[10] Bush L.P., Wilkinson H.H., Schardl C.L. (1997). Bioprotective alkaloids of grass-fungal endophyte symbioses. Plant Physiol..

[11] Rowan D.D., Hunt M.B., Gaynor D.L. (1986). Peramine, a novel insect feeding deterrent from ryegrass infected with the endophyte *Acremonium loliae*. J. Chem. Soc. Chem. Commun..

[12] Siegel M., Latch G., Bush L., Fannin F., Rowan D., Tapper B., Bacon C., Johnson M. (1990). Fungal endophyte-infected grasses: Alkaloid accumulation and aphid response. J. Chem. Ecol..

[13] Popay A., Hume D., Davis K., Tapper B. (2003). Interactions between endophyte (*Neotyphodium* spp.) and ploidy in hybrid and perennial ryegrass cultivars and their effects on Argentine stem weevil (*Listronotus bonariensis*). New Zealand J. Agric. Res..

[14] Fletcher L., Sutherland B. (2009). Sheep responses to grazing ryegrass with AR37 endophyte. Proc. New Zealand Grassl. Assoc..

[15] Fletcher L., Finch S., Sutherland B., deNicolo G., Mace W., Van Koten C., Hume D. (2017). The occurrence of ryegrass staggers and heat stress in sheep grazing ryegrass-endophyte associations with diverse alkaloid profiles. New Zealand Vet. J..

[16] Tapper B.A., Lane G.A. Janthitrems found in a Neotyphodium endophyte of perennial ryegrass. Proceedings of the 5th International Symposium on Neotyphodium/Grass Interactions.

[17] Finch S., Fletcher L., Babu J. (2011). The evaluation of endophyte toxin residues in sheep fat. New Zealand Vet. J..

[18] Ludlow E.J., Vassiliadis S., Ekanayake P.N., Hettiarachchige I.K., Reddy P., Sawbridge T.I., Rochfort S.J., Spangenberg G.C., Guthridge K.M. (2019). Analysis of the indole diterpene gene cluster for biosynthesis of the epoxy-janthitrems in *Epichloë* endophytes. Microorganisms.

[19] Babu J.V., Popay A.J., Miles C.O., Wilkins A.L., di Menna M.E., Finch S.C. (2018). Identification and structure elucidation of janthitrems A and D from Penicillium janthinellum and determination of the tremorgenic and anti-insect activity of janthitrems A and B. J. Agric. Food Chem..

[20] Hennessy L.M., Popay A.J., Finch S.C., Clearwater M.J., Cave V.M. (2016). Temperature and plant genotype alter alkaloid concentrations in ryegrass infected with an *Epichloë* endophyte and this affects an insect herbivore. Front. Plant Sci..

[21] Mantle P.G., Penn J. (1989). A role for paxilline in the biosynthesis of indole–diterpenoid penitrem mycotoxins. J. Chem. Soc. Perkin Trans. 1.

[22] Penn J., Mantle P.G. (1994). Biosynthetic intermediates of indole-diterpenoid mycotoxins from selected transformations at C-10 of paxilline. Phytochemistry.

[23] Young C.A., Tapper B.A., May K., Moon C.D., Schardl C.L., Scott B. (2009). Indole-diterpene biosynthetic capability of *Epichloë* endophytes as predicted by ltm gene analysis. Appl. Environ. Microbiol..

[24] Schardl C.L., Young C.A., Faulkner J.R., Florea S., Pan J. (2012). Chemotypic diversity of *Epichloë*, fungal symbionts of grasses. Fungal Ecol..

[25] Reddy P., Rochfort S., Read E., Deseo M., Jaehne E., Van Den Buuse M., Guthridge K., Combs M., Spangenberg G., Quinn J. (2019). Tremorgenic effects and functional metabolomics analysis of lolitrem B and its biosynthetic intermediates. Sci. Rep..

[26] Reddy P., Guthridge K., Vassiliadis S., Hemsworth J., Hettiarachchige I., Spangenberg G., Rochfort S. (2019). Tremorgenic mycotoxins: Structure diversity and biological activity. Toxins.

[27] Weedon C.M., Mantle P.G. (1987). Paxilline biosynthesis by *Acremonium loliae*; a step towards defining the origin of lolitrem neurotoxins. Phytochemistry.

[28] Gallagher R., Campbell A., Hawkes A., Holland P., McGaveston D., Pansier E., Harvey I. (1982). Ryegrass staggers: The presence of lolitrem neurotoxins in perennial ryegrass seed. New Zealand Vet. J..

[29] Miles C.O., Munday S.C., Wilkins A.L., Ede R.M., Towers N.R. (1994). Large-scale isolation of lolitrem B and structure determination of lolitrem E. J. Agric. Food Chem..

[30] Reddy P., Deseo M., Ezernieks V., Guthridge K., Spangenberg G., Rochfort S. (2019). Toxic indole diterpenes from endophyte-infected perennial ryegrass *Lolium perenne* L.: Isolation and stability. Toxins.

[31] Bailey P.T. (2007). Pests of Field Crops and Pastures: Identification and Control.

[32] Popay A.J., Cox N.R. (2016). *Aploneura lentisci* (Homoptera: Aphididae) and its interactions with fungal endophytes in perennial ryegrass (*Lolium perenne*). Front. Plant Sci..

[33] Hume D., Ryan D., Cooper B., Popay A. (2007). Agronomic performance of AR37-infected ryegrass in northern New Zealand. Proc. Conf.-New Zealand Grassl. Assoc..

[34] Popay A., Gerard P. (2007). Cultivar and endophyte effects on a root aphid *Aploneura lentisci* in perennial ryegrass. New Zealand Plant Prot..

[35] Vassiliadis S., Elkins A.C., Reddy P., Guthridge K.M., Spangenberg G.C., Rochfort S.J. (2019). A simple LC–MS method for the quantitation of alkaloids in endophyte-infected perennial ryegrass. Toxins.

[36] Peters F.T., Drummer O.H., Musshoff F. (2007). Validation of new methods. Forensic Sci. Int..

[37] Rasmussen S., Lane G.A., Mace W., Parsons A.J., Fraser K., Xue H. (2011). The use of genomics and metabolomics methods to quantify fungal endosymbionts and alkaloids in grasses. Plant Metabolomics. Methods in Molecular Biology.

[38] Spiering M.J., Davies E., Tapper B.A., Schmid J., Lane G.A. (2002). Simplified extraction of ergovaline and peramine for analysis of tissue distribution in endophyte-infected grass tillers. J. Agric. Food Chem..

[39] Zaiontz C. (2020). Real Statistics Using Excel.

[40] Smith D., Shappell N. (2002). Epimerization of ergopeptine alkaloids in organic and aqueous solvents. J. Anim. Sci..

[41] Hafner M., Sulyok M., Schuhmacher R., Crews C., Krska R. (2008). Stability and epimerisation behaviour of ergot alkaloids in various solvents. World Mycotoxin J..

[42] Lea K., Smith L., Gaskill C., Coleman R., Smith S. (2014). Ergovaline stability in tall fescue based on sample handling and storage methods. Front. Chem..

[43] Craig A.M., Bilich D., Hovermale J.T., Welty R.E. (1994). Improved extraction and HPLC methods for ergovaline from plant material and rumen fluid. J. Vet. Diagn. Investig..

[44] Rottinghaus G.E., Garner G.B., Cornell C.N., Ellis J.L. (1991). HPLC method for quantitating ergovaline in endophyte-infested tall fescue: Seasonal variation of ergovaline levels in stems with leaf sheaths, leaf blades, and seed heads. J. Agric. Food Chem..

[45] Ball O.-P., Barker G., Prestidge R., Lauren D. (1997). Distribution and accumulation of the alkaloid peramine in *Neotyphodium lolii*-infected perennial ryegrass. J. Chem. Ecol..

[46] Hewitt K.G., Mace W.J., McKenzie C.M., Matthew C., Popay A.J. (2020). Fungal alkaloid occurrence in endophyte-infected perennial ryegrass during seedling establishment. J. Chem. Ecol..

[47] Ruppert K.G., Matthew C., McKenzie C.M., Popay A.J. (2017). Impact of *Epichloë* endophytes on adult Argentine stem weevil damage to perennial ryegrass seedlings. Entomol. Exp. Et Appl..

[48] Popay A.J., Hume D.E., Mace W.J., Faville M.J., Finch S.C., Cave V. (2021). A root aphid *Aploneura lentisci* is affected by *Epichloë* endophyte strain and impacts perennial ryegrass growth in the field. Crop Pasture Sci..

[49] Repussard C., Zbib N., Tardieu D., Guerre P. (2014). Ergovaline and lolitrem B concentrations in perennial ryegrass in field culture in southern France: Distribution in the plant and impact of climatic factors. J. Agric. Food Chem..

[50] Rowan D.D., Dymock J.J., Brimble M.A. (1990). Effect of fungal metabolite peramine and analogs on feeding and development of Argentine stem weevil (*Listronotus bonariensis*). J. Chem. Ecol..

[51] Kanehisa M. (2019). Toward understanding the origin and evolution of cellular organisms. Protein Sci..

[52] Kanehisa M., Furumichi M., Sato Y., Ishiguro-Watanabe M., Tanabe M. (2021). KEGG: Integrating viruses and cellular organisms. Nucleic Acids Res..

[53] Kanehisa M., Goto S. (2000). KEGG: Kyoto encyclopedia of genes and genomes. Nucleic Acids Res..

[54] Springer J.P., Clardy J. (1980). Paspaline and paspalicine, two indole-mevalonate metabolites from *Claviceps paspali*. Tetrahedron Lett..

[55] Munday-Finch S.C. (1997). Aspects of the chemistry and toxicology of indole-diterpenoid mycotoxins involved in tremorganic disorder of livestock. Mycotoxin Res..

[56] Eady C. (2021). The impact of alkaloid-producing *Epichloë* endophyte on forage ryegrass breeding: A New Zealand perspective. Toxins.

[57] Gardner D.R., Welch K.D., Lee S.T., Cook D., Riet-Correa F. (2018). Tremorgenic indole diterpenes from *Ipomoea asarifolia* and *Ipomoea muelleri* and the identification of 6, 7-dehydro-11-hydroxy-12, 13-epoxyterpendole A. J. Nat. Prod..

[58] Popay A., Thom E. (2009). Endophyte effects on major insect pests in Waikato dairy pasture. Proc. New Zealand Grassl. Assoc..

[59] Pennell C., Popay A., Ball O.J.P., Hume D., Baird D. (2005). Occurrence and impact of pasture mealybug (*Balanococcus poae*) and root aphid (*Aploneura lentisci*) on ryegrass (*Lolium* spp.) with and without infection by *Neotyphodium* fungal endophytes. New Zealand J. Agric. Res..

