# Supplementary material for: Quantitation and Distribution of Epichloë-Derived Alkaloids in Perennial Ryegrass Tissues
Source: Metabolites. 2023 Jan 30;13(2):205. doi: 10.3390/metabo13020205 (PMC9966479; doi:10.3390/metabo13020205)
Supplement: Supplementary file 1 [file metabolites-13-00205-s001.zip › metabolites-2124012-supplementary.pdf]

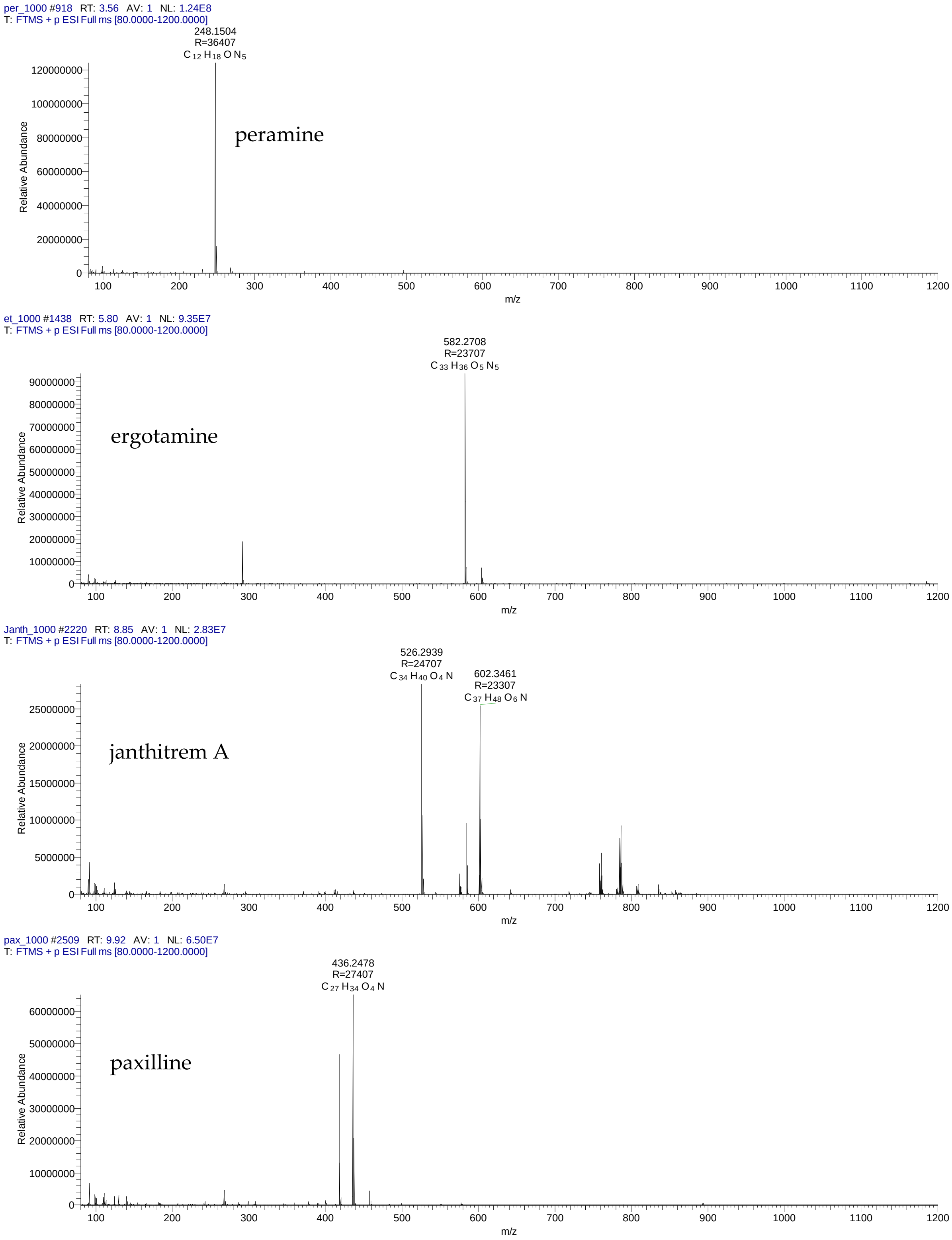

Figure S1. Positive MS1 spectra for the alkaloids. The spectra show that the fragment ions of *m/z* 526 (janthitrem A), *m/z* 420 (terpendole E) and *m/z* 436 (terpendole C) are of greater abundance compared to the parent ions (janthitrem A, *m/z* 602; terpendole E, *m/z* 438 and terpendole C, *m/z* 520, respectively). Both the parent and fragment ions were used for quantitative studies for comparative purposes.

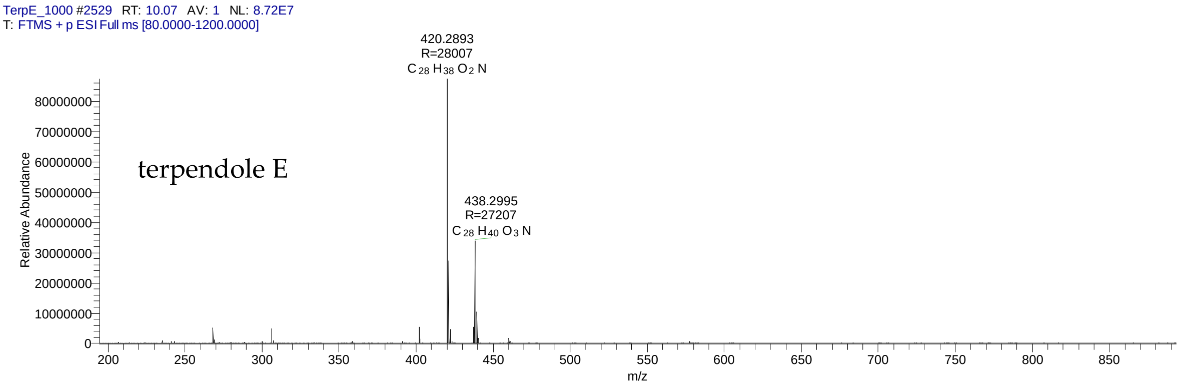

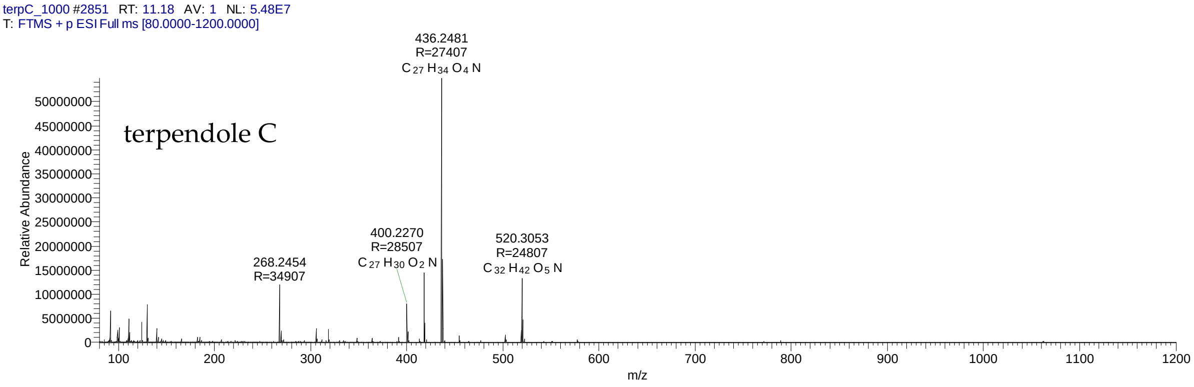

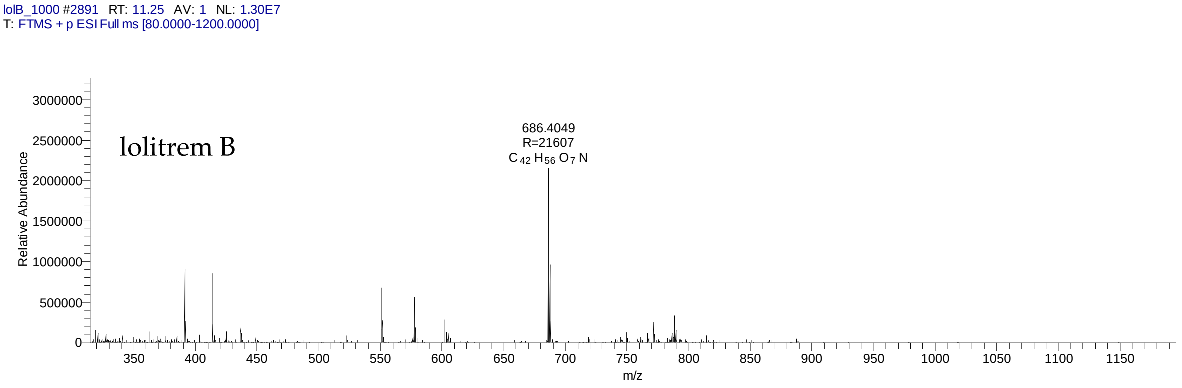

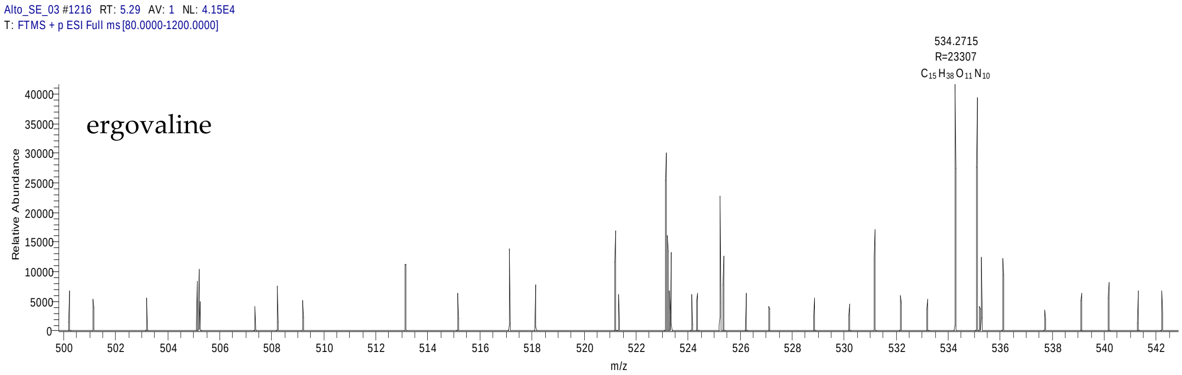

Figure S1. *Continue.*

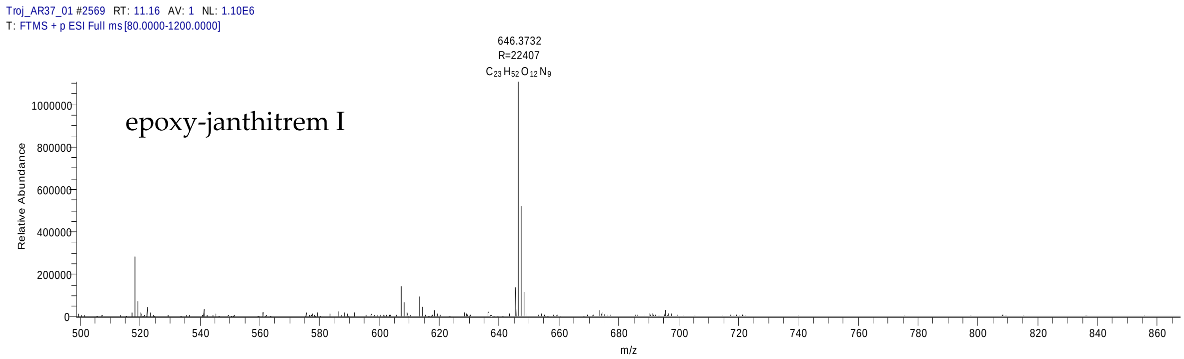

Figure S1. *Continue.*

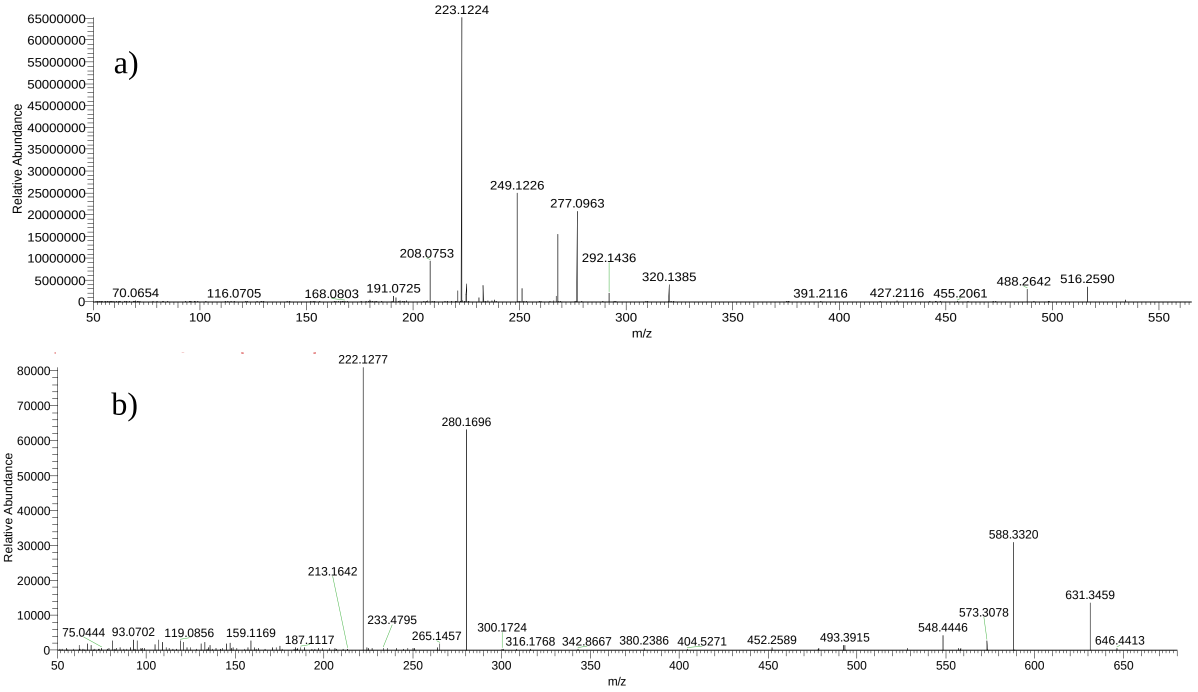

Figure S2. MS² fragmentation pattern of a) ergovaline and b) epoxy-janthitrem I.

Table S1. Recovery and matrix effect data for alkaloids pre- or post-spiked at a low, medium or high concentration in ryegrass shoots without endophyte (Alto-WE) or with endophyte (Alto-SE and Alto-NEA12). The alkaloids are peramine (per), ergovaline (ev), ergotamine (et), janthitrem A (janth A), paxilline (pax), terpendole E (terp E), epoxy-janthitrem I (janth I), terpendole C (terp C) and lolitrem B (lol B). The Mean values (peak area) and standard deviations (SD.) are in arbitrary units, x10^4. %RSD, percent relative standard deviation.

					per	ev	et	janth A[1]	janth A[2]	pax	terp E[1]	terp E[2]	janth I	terp C[1]	terp C[2]	lol B
Alto-WE	Shoots	Pre-spike	Low	Mean	511	na	197	47	85	115	54	212	na	9	92	26
				SD.	13	na	13	3	6	6	3	12	na	1	8	14
				%RSD	3	na	7	7	7	5	5	6	na	14	8	53
		Post-spike	Low	Mean	518	na	212	49	97	120	57	220	na	7	96	21
				SD.	8	na	6	1	2	5	3	10	na	2	6	10
				%RSD	1	na	3	1	2	4	5	5	na	29	6	46
		Pre-spike	Med	Mean	4590	na	1998	469	684	1132	548	1976	na	166	899	896
				SD.	158	na	109	22	14	43	25	99	na	13	59	39
				%RSD	3	na	5	5	2	4	5	5	na	8	7	4
		Post-spike	Med	Mean	4762	na	2221	539	803	1285	609	2182	na	188	974	952
				SD.	128	na	151	23	35	59	20	109	na	6	32	45
				%RSD	3	na	7	4	4	5	3	5	na	3	3	5
		Pre-spike	High	Mean	38986	na	24570	5133	6230	10528	5810	17978	na	1955	7663	9439
				SD.	585	na	1330	201	278	347	249	723	na	138	226	275
				%RSD	2	na	5	4	4	3	4	4	na	7	3	3
		Post-spike	High	Mean	44286	na	30428	8050	9569	17448	9129	25632	na	3175	12192	11633
				SD.	549	na	1472	223	345	516	344	1407	na	187	593	1074
				%RSD	1	na	5	3	4	3	4	5	na	6	5	9

Table S1. *Continued.*

					per	ev	et	janth A[1]	janth A[2]	pax	terp E[1]	terp E[2]	janth I	terp C[1]	terp C[2]	lol B
Alto-WE	Roots	Pre-spike	Low	Mean	352	na	175	65	114	177	77	291	na	12	139	106
				SD.	13	na	8	2	3	7	4	12	na	4	5	5
				%RSD	4	na	4	3	2	4	5	4	na	30	4	4
		Post-spike	Low	Mean	691	na	216	77	134	196	85	327	na	13	153	116
				SD.	22	na	8	3	5	10	2	9	na	3	6	5
				%RSD	3	na	3	4	3	5	3	3	na	22	4	4
		Pre-spike	Med	Mean	3476	na	2084	763	1071	1935	863	3140	na	316	1491	1188
				SD.	55	na	67	16	39	72	51	91	na	22	21	24
				%RSD	2	na	3	2	4	4	6	3	na	7	1	2
		Post-spike	Med	Mean	6024	na	2370	829	1147	2035	888	3300	na	317	1576	1281
				SD.	194	na	161	43	51	98	47	115	na	15	63	76
				%RSD	3	na	7	5	4	5	5	3	na	5	4	6
		Pre-spike	High	Mean	31343	na	28764	9341	10605	20550	10670	30438	na	4029	15190	12385
				SD.	256	na	1058	183	331	263	330	592	na	172	379	472
				%RSD	1	na	4	2	3	1	3	2	na	4	2	4
		Post-spike	High	Mean	50229	na	32704	10105	11451	21949	11562	32004	na	4258	15497	12480
				SD.	637	na	1403	203	242	461	215	898	na	111	256	262
				%RSD	1	na	4	2	2	2	2	3	na	3	2	2

Table S1. *Continued*.

					per	ev	et	janth A[1]	janth A[2]	pax	terp E[1]	terp E[2]	janth I	terp C[1]	terp C[2]	lol B
Alto-WE	Seed	Pre-spike	Low	Mean	534	na	181	63	110	180	76	286	na	21	134	103
				SD.	15	na	10	3	6	4	4	9	na	2	6	4
				%RSD	3	na	6	4	5	2	5	3	na	7	4	3
		Post-spike	Low	Mean	613	na	214	73	122	193	82	309	na	23	147	111
				SD.	28	na	21	2	4	7	4	7	na	3	6	5
				%RSD	5	na	10	3	3	3	4	2	na	15	4	4
		Pre-spike	Med	Mean	4893	na	1962	704	992	1878	829	2909	na	281	1352	1122
				SD.	61	na	60	25	39	42	44	96	na	17	46	49
				%RSD	1	na	3	4	4	2	5	3	na	6	3	4
		Post-spike	Med	Mean	5232	na	2227	727	1043	1949	858	2877	na	304	1450	1194
				SD.	174	na	243	54	45	72	49	280	na	19	55	149
				%RSD	3	na	11	7	4	4	6	10	na	6	4	13
		Pre-spike	High	Mean	45905	na	30277	8785	9915	20130	10602	29326	na	3550	13998	11645
				SD.	3496	na	2519	1343	1215	1408	720	1842	na	361	1167	956
				%RSD	8	na	8	15	12	7	7	6	na	10	8	8
		Post-spike	High	Mean	48108	na	31924	9781	11206	21452	10923	30957	na	3934	15052	12261
				SD.	710	na	1136	171	209	275	90	1313	na	117	524	497
				%RSD	1	na	4	2	2	1	1	4	na	3	3	4

Table S1. *Continued.*

					per	ev	et	janth A[1]	janth A[2]	pax	terp E[1]	terp E[2]	janth I	terp C[1]	terp C[2]	lol B
Alto-SE	Shoot	No	Endogenous	Mean	20586	57	na	na	na	6	6	37	na	5	153	378
				SD.	301	2	na	na	na	1	2	3	na	3	10	12
				%RSD	1	3	na	na	na	24	26	7	na	68	7	3
		Pre	Low	Mean	21529	44	168	44	80	117	55	223	na	16	185	390
				SD.	344	3	7	1	2	5	2	8	na	3	11	14
				%RSD	2	6	4	3	3	4	3	3	na	19	6	4
		Post	Low	Mean	21087	47	193	54	94	123	62	244	na	13	192	418
				SD.	95	2	7	5	5	22	5	15	na	2	10	22
				%RSD	0	4	3	9	5	18	8	6	na	13	5	5
		Pre	Med	Mean	23863	42	1800	435	625	985	472	1817	na	149	871	1249
				SD.	213	1	39	11	17	13	21	65	na	9	31	62
				%RSD	1	3	2	2	3	1	5	4	na	6	4	5
		Post	Med	Mean	24506	48	2012	563	805	1304	610	2248	na	200	1096	1345
				SD.	596	2	65	31	37	102	44	135	na	25	70	49
				%RSD	2	5	3	5	5	8	7	6	na	12	6	4
		Pre	High	Mean	56246	41	22877	4249	5207	8609	4793	15578	na	1484	6294	9466
				SD.	246	3	487	58	44	106	87	221	na	44	97	220
				%RSD	0	6	2	1	1	1	2	1	na	3	2	2
		Post	High	Mean	64067	49	29800	8131	9563	17198	8958	27056	na	3188	12661	12523
				SD.	724	1	1104	231	265	506	417	562	na	167	530	486
				%RSD	1	3	4	3	3	3	5	2	na	5	4	4

Table S1. *Continued.*

					per	ev	et	janth A[1]	janth A[2]	pax	terp E[1]	terp E[2]	janth I	terp C[1]	terp C[2]	lol B
Alto-SE	Roots	No	Endogenous	Mean	308	42	na	na	na	6	na	na	na	na	24	64
				SD.	20	3	na	na	na	1	na	na	na	na	1	6
				%RSD	6	6	na	na	na	23	na	na	na	na	4	9
		Pre	Low	Mean	599	36	185	67	118	192	78	301	na	12	168	174
				SD.	17	1	7	2	6	5	5	12	na	5	6	6
				%RSD	3	3	4	3	5	3	6	4	na	45	3	4
		Post	Low	Mean	874	38	228	76	127	213	84	330	na	11	187	191
				SD.	12	1	7	3	3	6	3	5	na	7	8	13
				%RSD	1	3	3	4	2	3	3	2	na	63	4	7
		Pre	Med	Mean	3161	35	1992	713	1015	1852	810	2972	na	294	1497	1261
				SD.	23	2	39	20	26	10	43	86	na	14	52	52
				%RSD	1	4	2	3	3	1	5	3	na	5	3	4
		Post	Med	Mean	5510	39	2416	780	1114	2045	883	3238	na	331	1606	1358
				SD.	196	3	134	47	53	108	51	230	na	31	70	71
				%RSD	4	7	6	6	5	5	6	7	na	9	4	5
		Pre	High	Mean	29095	36	27605	8894	10339	20543	10548	29744	na	3893	14879	12337
				SD.	430	2	682	215	245	275	238	821	na	114	506	473
				%RSD	1	4	2	2	2	1	2	3	na	3	3	4
		Post	High	Mean	47012	39	32915	9753	11296	22232	11821	32170	na	4239	16158	13260
				SD.	958	1	534	161	136	348	265	709	na	122	191	298
				%RSD	2	3	2	2	1	2	2	2	na	3	1	2

Table S1. *Continued.*

					per	ev	et	janth A[1]	janth A[2]	pax	terp E[1]	terp E[2]	janth I	terp C[1]	terp C[2]	lol B
Alto-SE	Seed	No	Endogenous	Mean	20690	3800	na	na	na	381	112	499	na	1334	6591	1417
				SD.	119	58	na	na	na	10	7	9	na	66	112	68
				%RSD	1	2	na	na	na	3	6	2	na	5	2	5
		Pre	Low	Mean	21535	3504	176	71	107	531	181	726	na	1199	6000	1355
				SD.	205	131	4	3	3	15	3	18	na	45	214	90
				%RSD	1	4	2	4	3	3	2	2	na	4	4	7
		Post	Low	Mean	22368	3754	201	78	117	573	205	801	na	1401	6705	1529
				SD.	684	196	14	5	5	14	11	8	na	97	269	25
				%RSD	3	5	7	6	4	2	5	1	na	7	4	2
		Pre	Med	Mean	24963	3485	1957	742	966	2209	936	3175	na	1544	7158	2334
				SD.	220	69	20	16	18	55	31	60	na	63	119	37
				%RSD	1	2	1	2	2	2	3	2	na	4	2	2
		Post	Med	Mean	26100	3660	2163	802	1036	2347	1010	3417	na	1714	7905	2557
				SD.	549	123	130	29	42	77	42	162	na	54	180	65
				%RSD	2	3	6	4	4	3	4	5	na	3	2	3
		Pre	High	Mean	61967	3459	28254	8722	9851	19653	10321	28256	na	4955	18986	12328
				SD.	587	104	753	84	117	243	434	616	na	213	318	297
				%RSD	1	3	3	1	1	1	4	2	na	4	2	2
		Post	High	Mean	66180	3678	31569	9837	11119	21921	11391	31124	na	5659	21235	13504
				SD.	952	135	1171	276	295	518	332	401	na	90	522	375
				%RSD	1	4	4	3	3	2	3	1	na	2	2	3

Table S1. *Continued.*

					per	ev	et	janth A[1]	janth A[2]	pax	terp E[1]	terp E[2]	janth I	terp C[1]	terp C[2]	lol B
Alto-NEA12	Shoots	No	Endogenous	Mean	na	na	na	na	na	7	8	40	710	na	10	na
				SD.	na	na	na	na	na	1	1	3	89	na	1	na
				%RSD	na	na	na	na	na	18	14	8	12	na	9	na
		Pre	Low	Mean	525	na	182	42	81	81	54	223	412	7	93	25
				SD.	17	na	5	3	2	14	1	13	23	1	9	11
				%RSD	3	na	3	7	3	17	2	6	6	21	9	42
		Post	Low	Mean	571	na	220	54	99	142	69	269	410	8	111	39
				SD.	7	na	5	3	3	8	6	13	15	3	5	13
				%RSD	1	na	2	6	3	6	9	5	4	40	5	32
		Pre	Med	Mean	4881	na	1975	441	648	1055	509	1891	426	158	809	850
				SD.	103	na	56	10	22	32	11	58	26	16	38	36
				%RSD	2	na	3	2	3	3	2	3	6	10	5	4
		Post	Med	Mean	5213	na	2211	541	796	1299	614	2257	443	187	1004	922
				SD.	84	na	43	20	20	28	39	58	17	15	22	28
				%RSD	2	na	2	4	2	2	6	3	4	8	2	3
		Pre	High	Mean	40555	na	24603	4631	5649	9509	5221	16687	348	1627	6932	9257
				SD.	823	na	1175	156	203	153	142	820	5	45	202	227
				%RSD	2	na	5	3	4	2	3	5	2	3	3	2
		Post	High	Mean	47361	na	29580	7903	9287	16894	8838	26201	443	3012	12211	11127
				SD.	264	na	956	199	214	417	397	744	15	67	415	215
				%RSD	1	na	3	3	2	2	4	3	3	2	3	2

Table S1. *Continued.*

					per	ev	et	janth A[1]	janth A[2]	pax	terp E[1]	terp E[2]	janth I	terp C[1]	terp C[2]	lol B
Alto-NEA12	Roots	No	Endogenous	Mean	na	na	na	na	na	5	2	4	810	na	2	na
				SD.	na	na	na	na	na	2	0	1	60	na	1	na
				%RSD	na	na	na	na	na	44	18	15	7	na	50	na
		Pre	Low	Mean	314	na	176	69	123	185	78	303	684	16	146	110
				SD.	10	na	10	2	6	11	7	16	37	4	8	7
				%RSD	3	na	5	3	5	6	8	5	5	23	5	7
		Post	Low	Mean	599	na	230	77	137	204	89	338	700	9	166	125
				SD.	7	na	11	1	2	9	5	12	151	5	9	11
				%RSD	1	na	5	1	1	4	5	3	22	55	5	9
		Pre	Med	Mean	3103	na	1962	762	1092	1897	829	2971	769	321	1419	1167
				SD.	81	na	95	36	56	69	30	130	57	23	65	60
				%RSD	3	na	5	5	5	4	4	4	7	7	5	5
		Post	Med	Mean	5285	na	2426	837	1197	2102	901	3330	770	316	1598	1281
				SD.	110	na	48	25	28	64	33	93	35	9	43	37
				%RSD	2	na	2	3	2	3	4	3	5	3	3	3
		Pre	High	Mean	29846	na	27504	9076	10746	20267	10615	29530	784	3995	15012	12351
				SD.	690	na	1095	238	232	442	449	280	23	198	435	284
				%RSD	2	na	4	3	2	2	4	1	3	5	3	2
		Post	High	Mean	47456	na	32172	9709	11557	21989	11442	32074	786	4186	15808	12941
				SD.	706	na	1167	181	260	564	198	1329	31	112	530	718
				%RSD	1	na	4	2	2	3	2	4	4	3	3	6

Table S1. *Continued.*

					per	ev	et	janth A[1]	janth A[2]	pax	terp E[1]	terp E[2]	janth I	terp C[1]	terp C[2]	lol B
Alto-NEA12	Seed	No	Endogenous	Mean	na	na	na	na	na	153	41	186	4280	na	64	na
				SD.	na	na	na	na	na	13	4	7	835	na	18	na
				%RSD	na	na	na	na	na	8	10	4	20	na	28	na
		Pre	Low	Mean	501	na	174	63	106	313	113	443	4219	25	159	100
				SD.	19	na	9	5	5	7	5	23	352	3	12	8
				%RSD	4	na	5	8	5	2	4	5	8	13	7	8
		Post	Low	Mean	591	na	219	74	122	345	132	485	4647	29	182	108
				SD.	14	na	17	1	7	13	8	19	390	3	3	3
				%RSD	2	na	8	2	6	4	6	4	8	9	2	3
		Pre	Med	Mean	4663	na	1859	656	914	1863	783	2847	4092	264	1328	1025
				SD.	90	na	76	21	24	42	25	96	57	11	39	39
				%RSD	2	na	4	3	3	2	3	3	1	4	3	4
		Post	Med	Mean	5360	na	2185	748	1046	2118	879	3222	4469	299	1512	1131
				SD.	140	na	166	32	41	75	33	133	245	25	56	36
				%RSD	3	na	8	4	4	4	4	4	5	9	4	3
		Pre	High	Mean	43036	na	28198	8549	9625	19166	9774	27959	4322	3438	13551	11215
				SD.	402	na	933	239	212	405	296	886	347	94	237	241
				%RSD	1	na	3	3	2	2	3	3	8	3	2	2
		Post	High	Mean	47891	na	31372	9918	11164	21362	10946	30917	4786	3918	14999	11924
				SD.	135	na	1412	73	106	578	200	562	314	120	348	462
				%RSD	0	na	4	1	1	3	2	2	7	3	2	4

Table S2. Comparison of the mean alkaloid concentrations in different ryegrass-endophyte associations (Alto-SE, Alto-NEA12) and quantitated using the calibration curves constructed by in-standard matrixes (shoots, roots or seeds).

calibration curve	plant	tissue	stat	per	ev	pax	terp E[1]	terp E[2]	janth I[1]	janth I[2]	terp C[1]	terp C[2]	lol B
shoots matrix	Alto-SE	Shoot	mean	42.26	0.27	0.04	0.06	0.14	0	0	0.15	1.26	3.90
			SEM	0.65	0.01	0.01	0.02	0.01	0	0	0.10	0.08	0.12
			% RSD	1.53	2.96	24.10	26.11	7.01	0	0	68.24	6.58	3.07
shoots matrix	Alto-SE	Root	mean	0.63	0.15	0.04	0	0	0	0	0	0.20	0.66
			SEM	0.04	0.01	0.01	0	0	0	0	0	0.01	0.06
			% RSD	6.49	6.14	22.41	0	0	0	0	0	4.10	8.84
shoots matrix	Alto-SE	Seed	mean	42.30	13.49	2.40	1.17	1.84	0	0	41.97	53.90	14.57
			SEM	0.26	0.23	0.07	0.07	0.03	0	0	2.00	0.94	0.70
			% RSD	0.61	1.70	2.84	6.20	1.57	0	0	4.76	1.75	4.78
shoots matrix	Alto-NEA12	Shoot	mean	0	0	0.05	0.09	0.15	11.91	8.49	0	0.08	0
			SEM	0	0	0.01	0.01	0.01	1.50	1.07	0	0.01	0
			% RSD	0	0	17.83	13.59	7.97	12.57	12.57	0	8.85	0
shoots matrix	Alto-NEA12	Root	mean	0	0	0.03	0.02	0.02	13.56	9.66	0	0.02	0
			SEM	0	0	0.01	0.00	0.00	1.04	0.74	0	0.01	0
			% RSD	0	0	43.51	18.01	15.16	7.69	7.69	0	50.42	0
shoots matrix	Alto-NEA12	Seed	mean	0	0	0.96	0.42	0.69	71.65	51.04	0	0.52	0
			SEM	0	0	0.08	0.04	0.02	14.03	10.00	0	0.15	0
			% RSD	0	0	8.32	10.49	3.10	19.58	19.58	0	28.09	0
roots matrix	Alto-SE	Shoot	mean	37.21	0.27	0.03	0.05	0.11	0	0	0.01	0.87	3.17
			SEM	0.57	0.01	0.01	0.01	0.01	0	0	0.01	0.06	0.10
			% RSD	1.53	2.96	24.10	26.11	7.01	0	0	68.24	6.58	3.07
roots matrix	Alto-SE	Root	mean	0.55	0.15	0.03	0	0	0	0	0	0.14	0.54

calibration curve	plant	tissue	stat	per	ev	pax	terp E[1]	terp E[2]	janth I[1]	janth I[2]	terp C[1]	terp C[2]	lol B
			SEM	0.04	0.01	0.01	0	0	0	0	0	0.01	0.05
			% RSD	6.49	6.14	22.41	0	0	0	0	0	4.10	8.84
roots matrix	Alto-SE	Seed	mean	37.25	13.49	1.90	0.92	1.47	0	0	3.66	37.38	11.85
			SEM	0.23	0.23	0.05	0.06	0.02	0	0	0.17	0.65	0.57
			% RSD	0.61	1.70	2.84	6.20	1.57	0	0	4.76	1.75	4.78
roots matrix	Alto-NEA12	Shoot	mean	0	0	0.04	0.07	0.12	11.91	8.49	0	0.06	0
			SEM	0	0	0.01	0.01	0.01	1.50	1.07	0	0.01	0
			% RSD	0	0	17.83	13.59	7.97	12.57	12.57	0	8.85	0
roots matrix	Alto-NEA12	Root	mean	0	0	0.03	0.02	0.01	13.56	9.66	0	0.01	0
			SEM	0	0	0.01	0.00	0.00	1.04	0.74	0	0.01	0
			% RSD	0	0	43.51	18.01	15.16	7.69	7.69	0	50.42	0
roots matrix	Alto-NEA12	Seed	mean	0	0	0.76	0.33	0.55	71.65	51.04	0	0.36	0
			SEM	0	0	0.06	0.03	0.02	14.03	10.00	0	0.10	0
			% RSD	0	0	8.32	10.49	3.10	19.58	19.58	0	28.09	0
seed matrix	Alto-SE	Shoot	mean	38.90	0.27	0.03	0.05	0.11	0	0	0.12	1.01	3.70
			SEM	0.59	0.01	0.01	0.01	0.01	0	0	0.08	0.07	0.11
			% RSD	1.53	2.96	24.10	26.11	7.01	0	0	68.24	6.58	3.07
roots matrix	Alto-SE	Root	mean	0.58	0.20	0.03	0	0	0	0	0	0.16	0.63
			SEM	0.04	0.01	0.01	0	0	0	0	0	0.01	0.06
			% RSD	6.49	6.14	22.41	0	0	0	0	0	4.10	8.84
roots matrix	Alto-SE	Seed	mean	38.94	18.31	1.95	0.97	1.52	0	0	33.81	43.58	13.81
			SEM	0.24	0.31	0.06	0.06	0.02	0	0	1.61	0.76	0.66
			% RSD	0.61	1.70	2.84	6.20	1.57	0	0	4.76	1.75	4.78
roots matrix	Alto-NEA12	Shoot	mean	0	0	0.04	0.07	0.12	11.91	8.49	0	0.07	0
			SEM	0	0	0.01	0.01	0.01	1.50	1.07	0	0.01	0

calibration curve	plant	tissue	stat	per	ev	pax	terp E[1]	terp E[2]	janth I[1]	janth I[2]	terp C[1]	terp C[2]	lol B
			% RSD	0	0	17.83	13.59	7.97	12.57	12.57	0	8.85	0
roots matrix	Alto-NEA12	Root	mean	0	0	0.03	0.02	0.01	13.56	9.66	0	0.01	0
			SEM	0	0	0.01	0.00	0.00	1.04	0.74	0	0.01	0
			% RSD	0	0	43.51	18.01	15.16	7.69	7.69	0	50.42	0
roots matrix	Alto-NEA12	Seed	mean	0	0	0.78	0.35	0.57	71.65	51.04	0	0.42	0
			SEM	0	0	0.06	0.04	0.02	14.03	10.00	0	0.12	0
			% RSD	0	0	8.32	10.49	3.10	19.58	19.58	0	28.09	0

Alkaloids are peramine (per), ergovaline (ev), paxilline (pax), terpendole E (terp E), epoxy-janthitrem I (janth I), terpendole C (terp C) and lolitrem B (lol B). Concentrations are indicated in mg/kg. SEM, standard error of the mean, % RSD, percent relative standard deviation of five different extracts (n =5). [1]Parent ion in the MS1 spectrum; [2]Fragment ion in the MS1 spectrum. No alkaloids were detected in ryegrass plants without endophyte (WE).

Table S3. The effect of alkaloid concentration (low or high spikes) and plant matrix (shoot, root or seed) on mass accuracy.

				shoot		root		seed	
				low spike	high spike	low spike	high spike	low spike	high spike
**Alkaloid**	**Ion (*m/z*)**	**RT (min)**	**Formula [M+H]⁺**	**Δ ppm**	**Δ ppm**	**Δ ppm**	**Δ ppm**	**Δ ppm**	**Δ ppm**
peramine	248.1504	3.56	$C_{12}H_{18}N_5O$	0.62	0.82	-0.07	-0.11	-0.59	-0.91
ergotamine	582.2708	5.79	$C_{33}H_{36}N_5O_5$	0.04	0.56	0.88	0.04	0.15	-0.90
janthitrem A[1]	602.3461	8.85	$C_{37}H_{48}NO_6$	-1.15	-0.24	0.08	-0.84	-0.84	-1.15
janthitrem A[2]	526.2939	8.85	$C_{34}H_{40}NO_4$	-0.96	-0.16	-0.85	-0.39	-0.03	-0.73
paxilline	436.2478	9.92	$C_{27}H_{34}NO_4$	-2.33	0.70	0.26	-0.29	-0.08	-0.93
terpendole E[1]	438.2995	10.07	$C_{28}H_{40}NO_3$	-0.09	-0.09	-0.23	-0.23	-0.71	-1.35
terpendole E[2]	420.2894	10.07	$C_{28}H_{38}NO_2$	-0.16	0.13	0.30	-0.09	-0.87	-0.59
terpendole C[1]	520.3053	11.18	$C_{32}H_{42}NO_5$	-2.15	-0.29	-4.09	-0.52	0.77	-0.87
terpendole C[2]	436.2481	11.18	$C_{27}H_{34}NO_4$	-0.22	0.20	0.54	0.06	-0.22	-0.29
lolitrem B	686.4049	11.25	$C_{42}H_{56}NO_7$	2.39	-0.77	0.66	-0.06	-0.32	-0.41

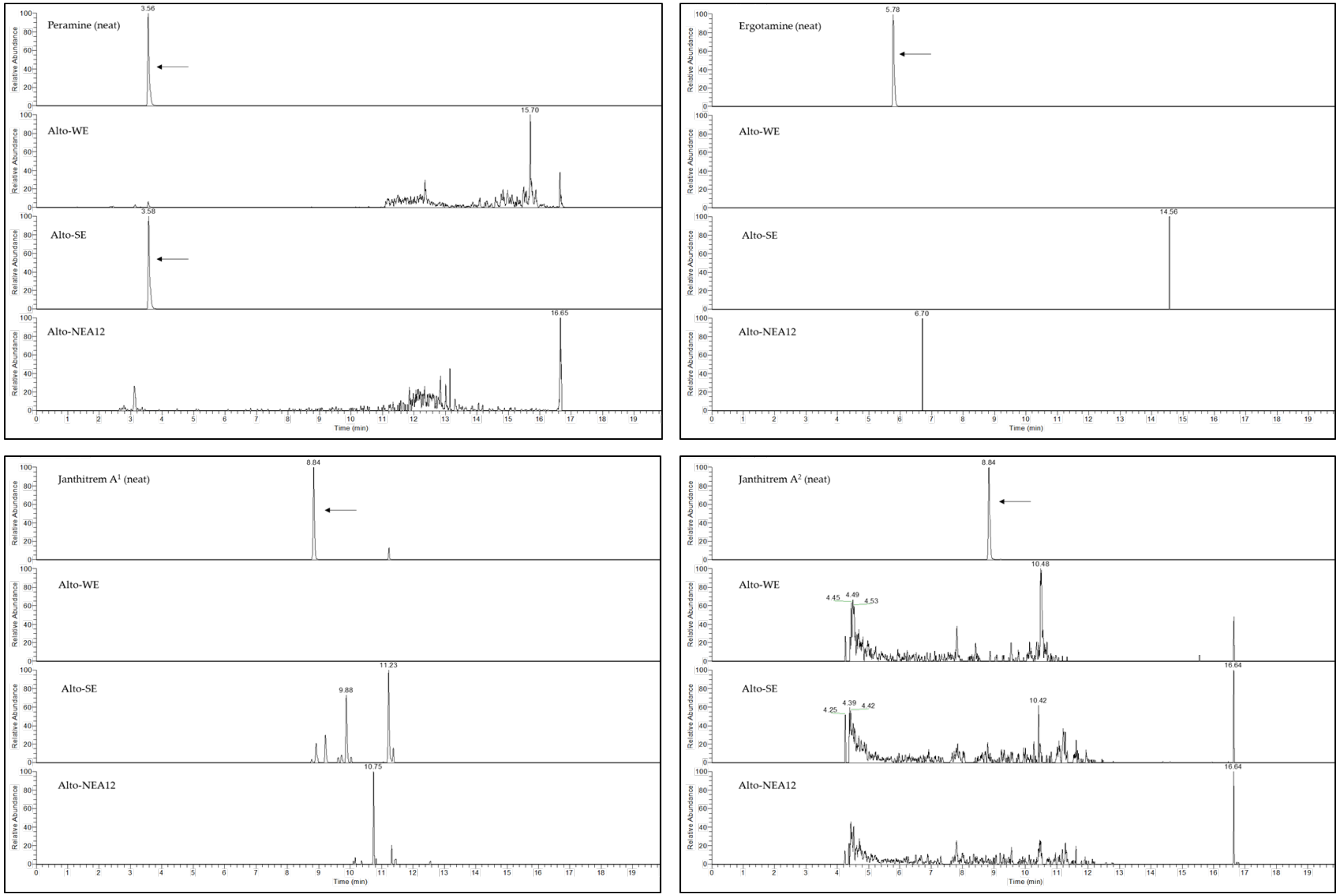

Figure S3. Extracted ion chromatograms (EIC) of alkaloids in neat solution and in Alto-WE, Alto-SE and Alto-NEA12 of a representative seed sample. Arrows show the peak at a given retention time.

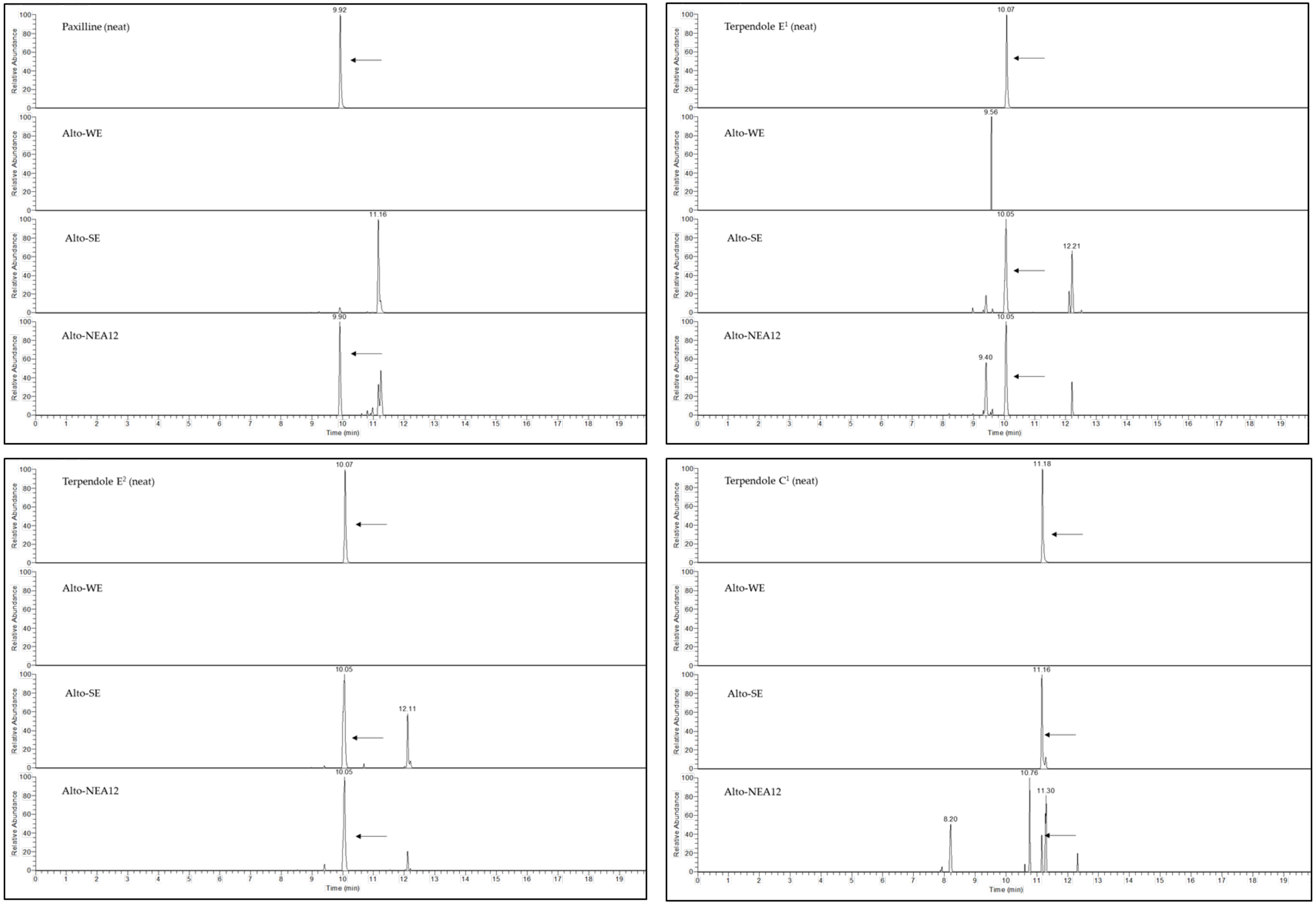

Figure S3. *Continued.*

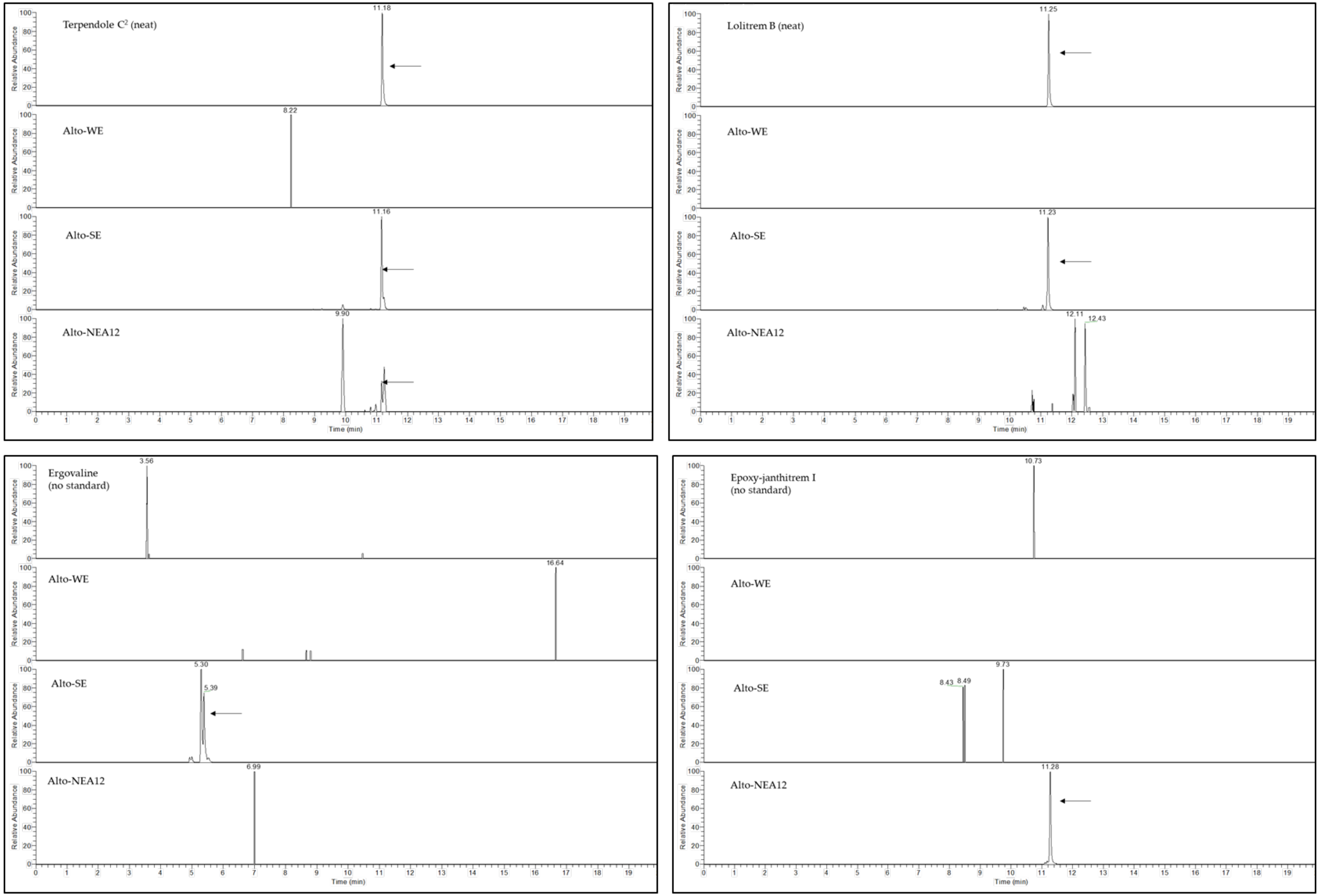

Figure S3. *Continued.*
